# Differentiation and on axon-guidance chip culture of human pluripotent stem cell-derived peripheral cholinergic neurons for airway neurobiology studies

**DOI:** 10.3389/fphar.2022.991072

**Published:** 2022-10-28

**Authors:** P. A. Goldsteen, A. M. Sabogal Guaqueta, P. P. M. F. A. Mulder, I. S. T. Bos, M. Eggens, L. Van der Koog, J. T. Soeiro, A. J. Halayko, K. Mathwig, L. E. M. Kistemaker, E. M. J. Verpoorte, A. M. Dolga, R. Gosens

**Affiliations:** ^1^ Department of Molecular Pharmacology, University of Groningen, Groningen, Netherlands; ^2^ GRIAC, Groningen Research Institute for Asthma and COPD, University of Groningen, Groningen, Netherlands; ^3^ Department of Pharmaceutical Analysis, University of Groningen, Groningen, Netherlands; ^4^ Department of Physiology and Pathophysiology, University of Manitoba, Winnipeg, MB, Canada; ^5^ Aquilo BV, Groningen, Netherlands

**Keywords:** neuron, asthma, organ-on-chip, cholinergic, stem cell

## Abstract

Airway cholinergic nerves play a key role in airway physiology and disease. In asthma and other diseases of the respiratory tract, airway cholinergic neurons undergo plasticity and contribute to airway hyperresponsiveness and mucus secretion. We currently lack human *in vitro* models for airway cholinergic neurons. Here, we aimed to develop a human *in vitro* model for peripheral cholinergic neurons using human pluripotent stem cell (hPSC) technology. hPSCs were differentiated towards vagal neural crest precursors and subsequently directed towards functional airway cholinergic neurons using the neurotrophin brain-derived neurotrophic factor (BDNF). Cholinergic neurons were characterized by ChAT and VAChT expression, and responded to chemical stimulation with changes in Ca^2+^ mobilization. To culture these cells, allowing axonal separation from the neuronal cell bodies, a two-compartment PDMS microfluidic chip was subsequently fabricated. The two compartments were connected *via* microchannels to enable axonal outgrowth. On-chip cell culture did not compromise phenotypical characteristics of the cells compared to standard culture plates. When the hPSC-derived peripheral cholinergic neurons were cultured in the chip, axonal outgrowth was visible, while the somal bodies of the neurons were confined to their compartment. Neurons formed contacts with airway smooth muscle cells cultured in the axonal compartment. The microfluidic chip developed in this study represents a human *in vitro* platform to model neuro-effector interactions in the airways that may be used for mechanistic studies into neuroplasticity in asthma and other lung diseases.

## Introduction

The lungs are innervated through a dense network of afferent and efferent nerves, which are arranged along the vagus nerve (van der Velden and Hulsmann, 1999). Among the efferent nerves, the parasympathetic neurons are most dominant in controlling several effector functions, including: airway smooth muscle (ASM) tone and mucus secretion (Kistemaker and Prakash, 2019). As is the case for all neurons, the airway nervous system is subjected to changes over time in response to intrinsic and extrinsic stimuli, known as neuronal plasticty (Nockher and Renz, 2006; Cramer et al., 2011). Severe or prolonged stimuli can cause permanent changes to the neurons, manifested as altered neurite length or innervation, lowered firing threshold, or even phenotype switching (Vasina et al., 2006; Undem and Taylor-Clark, 2014). Asthma patients have an increased innervation of both the sensory and the autonomic cholinergic nervous system (Drake et al., 2018; Dragunas et al., 2020). In asthma, neuroplasticity of the cholinergic nervous system is a newly discovered phenomenon (Dragunas et al., 2020). However, we do not yet fully understand the underlying mechanisms.

Conventional models, such as animal models or patient biopsies, can only provide limited information about mechanisms underlying neuroplasticity (Goldsteen et al., 2020). Patient biopsies provide much information on the final stages of neuronal remodeling. Animal models can be favored over biopsies for mechanistic studies as they take into account full physiological complexity, and the nervous system is well integrated into the organs and connected to the central nervous system. However, the problem with animal models is that the translation to the human situation is poor (Pound and Bracken, 2014; Leenaars et al., 2019). Human pluripotent stem cells (hPSCs) can aid in the development of a human disease model to study the process of neuronal plasticity *in vitro* (Goldsteen et al., 2020). Developing a human *in vitro* model to understand neuroplasticity in asthma has mostly been hampered by a lack of protocols for robust differentiation from hPSCs to that of airway cholinergic neurons.

Airway cholinergic neurons and enteric neurons originate from a vagal neural crest cell (NCC) precursor before developing into different directions (Aven and Ai, 2013; Hutchins et al., 2018). The differentiation of NCCs towards the peripheral neurons is dependent on neurotrophic factor (NTF) signaling. NTFs regulate neurogenesis, neuronal differentiation, neuronal survival, nerve conduction, and neuronal plasticity (Huang and Reichardt, 2001; Aven and Ai, 2013). Brain-derived neurotrophic factor (BDNF) is the predominant NTF in the lungs (Ricci et al., 2004), opposed to glial cell-derived neurotrophic factor (GDNF) in the enteric system (Fattahi et al., 2016; Barber et al., 2019). Secreted NTFs act as essential chemo-attractants for NCCs. For example, airway smooth muscle secretes BDNF in the lungs and guides airway neurons towards the muscle during development (Radzikinas et al., 2011).

Peripheral nervous system (PNS) neurons should be cultured and arranged correctly to anatomically reconstruct neuro-effector interactions *in vitro*. Several devices have been designed to study this axonal communication. The Campenot chamber is a Teflon-based culture chamber to separate neurites from cell bodies, used as the first compartmentalized culture technique to study the local effects of growth factors on neurite outgrowth (Campenot, 1977). As a follow-up, microfabrication techniques have been expanded into tailoring suitable devices for PNS-effector interactions (Goldsteen et al., 2020). Next-generation compartmentalized cell culture vessels were created from polydimethylsiloxane (PDMS), a biocompatible and versatile polymer that is easy to use and fabricate microfluidic chips even in submicron dimensions (Mata et al., 2005). PDMS chambers have been widely integrated into organ-on-a-chip devices, including compartmentalized axon-guidance chips (Taylor et al., 2005). This design has been adapted many times and is highly suitable for integrating PNS neurons and their target organs (Taylor et al., 2003; Takayama and Kida, 2016; Neto et al., 2014; Goldsteen et al., 2020).

In this study, we established a robust protocol for cholinergic neuron differentiation for studies on neuro-effector communication in the respiratory tract. Using dual SMAD inhibition and Wnt activation, p75^+^-HNK1^+^ NCC precursors were generated. Subsequently, vagal NCCs were guided into mature and functional peripheral cholinergic neurons using BDNF. To establish a platform on which these cells can be co-cultured with effector cells, we created a PDMS chip, consisting of two culture compartments, a somal and an axonal compartment, connected with tapered microchannels. In the somal compartment, we cultured neuronal cell bodies, whereas the axonal compartment displayed axonal outgrowth of the neurons and cell contact with airway smooth muscle cells. Cell morphology and function were evaluated on both regular plastic cell culture plates and the microfluidic chips, comparing microscopic observations, mRNA expression, immunofluorescence staining, and live-cell calcium imaging. We thus provide technical feasibility data for this culture system as a suitable device for PNS neuronal culture.

## Materials and methods

### H9WA09 cell culturing

H9WA09 cells were obtained from the European Institute for the Biology of Ageing (ERIBA) at the University of Groningen. H9WA09 cells were cultured on Matrigel hESC-qualified (Corning, 354277) pre-coated 6-well plates in mTeSR1 medium (STEMCELL technologies, 85850). The cells were incubated at 5% CO_2_ and 37°C. Once the cells grew confluent, the H9WA09 cells were passaged using ReLeSR (STEMCELL technologies, 05872). The pluripotency of the H9WA09 cells was tested regularly by staining for the pluripotency-marker OCT4. In addition, the cells were examined regularly for the presence of *mycoplasma*.

### Differentiating H9WA09 cells towards a neuronal cell fate

For differentiation of H9WA09 cells into airway cholinergic neurons, several stages were passed (Supplementary Table S1). Vagal NCC induction was started when pluripotent stem cells were 40–60% confluent. Pluripotent stem cells were first differentiated into vagal NCCs in 12 days. Two types of media were used: KSR medium (KnockOut DMEM (Thermo Fisher, 10829018) and 15% KnockOut Serum Replacement (Thermo Fisher, 10828028)) and N2 medium (DMEM HEPES (Thermo Fisher, 12320032), 1% Penicillin-Streptomycin (Thermo Fisher, 15070063), and 10 μg/mL N2 supplement (Thermo Fisher, A1370701)). The manufacturer supplies N2 supplement as 100x, but double the amount was used in this protocol. KSR and N2 medium were freshly supplemented with 10 µM SB431542 (STEMCELL Technologies, Vancouver, Canada, 72234), 1 µM LDN193189 (STEMCELL Technologies, Vancouver, Canada, 72147), 3 µM CHIR99021 (STEMCELL Technologies, Vancouver, Canada, 72054), and 1 µM retinoic acid. The medium was changed every other day, medium composition according to Supplementary Table S2.

Next, the vagal NCCs were cultured in the form of floating spheroids for 4 days. The cells were washed with EDTA (0.5 mM) twice, followed by 10 min incubation at 37°C. After aspirating the EDTA, vagal precursor (VP) spheroid medium was added, consisting of Neurobasal Medium (Thermo Fisher, 21103049) supplemented with 10 μL/mL N2 supplement, 20 μL/ml B27 supplement (Thermo Fisher, 17504044), 10 μL/ml Glutamax (Thermo Fisher, 35050061), and 10 μL/ml MEM Nonessential Amino Acids). Prior to medium change, 10 ng/ml FGF2 (Thermo Fisher, PHG6015) and 3 µM CHIR99021 (STEMCELL Technologies, 72054) was freshly added. The cells were mechanically detached in VP spheroid medium using a serological pipette before transfer to a 6-well plate pre-coated with anti-adherence solution (STEMCELL Technologies, 07010). Detached cells from five wells of a 6-well plate were added divided over six wells of a 6-well plate. On day 14, the medium was refreshed.

After spheroid formation, the vagal cells undergo airway cholinergic neuron induction and airway cholinergic neuron maturation. On day 16 of the protocol, the VP spheroid medium was aspirated, and the spheroids were dissociated using EDTA (0.5 mM; wash twice, followed by 10 min incubation, 37°C). The cell suspension was carefully transferred to a tube and centrifuged (290 g, 1 min, RT). The supernatant was aspirated, and the cell pellet was resuspended in airway neuron (AN) medium (Neurobasal medium supplemented with 10 μg/mL N2 supplement, 20 μL/ml B27 supplement, 10 μg/ml Glutamax, and 10 μg/ml MEM Nonessential Amino Acids). 10 ng/ml BDNF (Peprotech, 450-02) and 100 µM freshly prepared l-Ascorbic Acid (Sigma-Aldrich, A5960) were added before medium change. Dissociated spheroids were plated onto culture plates pre-coated with 15 μg/ml Poly-l-Ornithine (PLO, Sigma-Aldrich, P4538), 2 μg/ml fibronectin (FB, Thermo Fisher, 33016015), and 2 μg/ml laminin (LM, R&D systems, 3400-010-02).

In the first stage of airway cholinergic neuron induction (day 16–30), the medium was changed three times per week, changing ¾ of the total volume. In the second stage (day 30–40), the medium was changed twice per week the medium volume was 1.5 times increased. In addition, from day 35 onwards, AN medium was supplemented with 2 μg/ml FB and 2 μg/ml LM. In the final stage of airway cholinergic neuron maturation (day 40–50), the medium was changed once per week. Cells were stimulated with 10 nM dexamethasone, or with the cytokines IL-4 (10 ng/ml, Peprotech, 200-04), IL-13 (3 ng/ml, Peprotech, 200-13), and IL-33 (10 ng/ml, Peprotech, 200-33). PCR analysis and immunofluorescence (IF) staining were performed on different time points: day 25, 35, and day 50. Additionally, fluorescence-activated cell sorting (FACS) was performed on day 50.

### Gene expression analysis RT-qPCR

mRNA was isolated using NucleoSpin RNA XS kit (740902.50, Macherey-Nagel, Dueren, Germany) according to the manufacturer’s protocol. The yield and purity of the isolated RNA were measured using the NanoDrop 1000 spectrophotometer and further processed for RT-qPCR or RNA sequencing.

For RT-qPCR, cDNA was synthesized using Reverse Transcription System (A3500, Promega, Leiden, Netherlands) according to the manufacturer’s protocol. The qRT-PCR reactions were completed using SYBR Green. A list of the qPCR forward primers and reverse primers used is provided in Supplementary Table S4. The program for RT-qPCR reactions started with polymerase activation at 95°C for 10 min, 45 cycles of PCR cycling, which included denaturation at 95°C for 30 s, annealing for 30 s at 59°C, and extension at 72°C for 30 s, and incubation at 72°C for 5 min. Melting curves were obtained consecutively: 15 s at 95°C, 15 s at 55°C, and 15 s at 95°C. Analysis of the gene expression was performed with Quantstudio Real-Time PCR software v1.2.

RNA for sequencing was sent to GenomeScan BV (Leiden, Netherlands), and raw counts were provided together with a quality report. Genes with at least one count per million (CPM) were considered expressed and included for further analysis. Non-expressed genes were removed. Normalized expression levels of several neuronal and non-neuronal markers was compared. Normalized counts can be found in Supplementary Table S3 and on GEO using accession number GSE211478.

### Immunofluorescence staining of cultures

Cells were fixed in 4% paraformaldehyde (PFA, Sigma-Aldrich, 97H0752) and permeabilized using 0.3% Triton X (Sigma-Aldrich, 101371900) for 5 min, RT. The cells were blocked for 1 h with blocking buffer, consisting of Cyto-TBS + 2% bovine serum albumin (Sigma Aldrich, 1002695029) and directly after incubated with a primary antibody overnight at 4°C. See Supplementary Table S5 for used antibodies and dilutions. The next day, the cells were incubated with a secondary antibody for 2 h in the dark, RT. Optionally, cells were counterstained for 45 min using 1 unit/assay Alexa Fluor 488 Phalloidin (Thermo Fisher, A12379). Mounting medium with DAPI (Abcam, ab104139) was used. Samples were imaged using a TissueFAXS (TissueGnostics) or a Zeiss LSM 780 (Zeiss, Germany) microscope and analyzed using Fiji (http://fiji.sc/).

### Live cell CA^2+^ imaging using Fluo-4 AM

Live-cell imaging to show mature neuronal response to potassium chloride (KCl) or metacholine (MCh) was performed using Fluo-4-AM (Invitrogen™, F14217). Cells were incubated with Fluo-4-AM in HBSS-Ca^2+^ (45 min, RT, dark) and maintained at RT in the dark until data acquisition. Data were acquired using the Zeiss LSM 780 microscope. 3-10 neurons were identified for data acquisition. Cells were excited with 488-nm light (for Fluo-4-AM) and red-nm light (for 7-ADD cell death marker). Images were collected by taking an image every 100 msec for 3 min. After 30 s, the cells were challenged by adding 60 mM KCl or 100 μM MCh. To ensure that KCl enters the somal compartment within seconds, a volume of 10% of the chip volume was added (20 µl KCl solution into 200 µl HBSS). An increase in intensity was measured to quantify the neuronal response. A supplementary video is provided to demonstrate the calcium response (Supplementary Video S1).

### Spontaneous firing of neurons

A Maestro Pro (Axion Biosystems) multi-electrode array (MEA) system was used to measure the spontaneous firing of neurons (Trombetta-Lima et al., 2021). We coated CytoView MEA 48 plates (Axion Biosystems, M768-tMEA-48W) containing sixteen embedded electrodes per well with PO/LM/FN. The coating solution was aspirated, and the wells were left to dry for 30 min. Dissociated NCC spheroids (day 16) were seeded in high-concentration LM (10 μM/ml) drops (5 µl/drop) into the center of the MEA well. The cells were incubated (60 min, 37 °C) before adding AN medium. Repeated recordings were made every 10 days for 15 min. The MEA plate was inserted into the MEA Maestro (37C, 5% CO_2_) for spike detection. Axion AxIS Software recorded raw voltage data and detected spikes for rate analysis.

### Chip fabrication

The axon-guidance chip master was fabricated from PDMS using photo–and soft lithography according to Peyrin et al., 2011. We used an additional PDMS-PFPE molding step to increase the viability of the cells cultured in the chip. The replica molding technique was partly derived from Jellali et al., 2016 (Peyrin et al., 2011; Jellali et al., 2016). For producing the master mold, SU-8 2002 (micro resist technology GmbH, Berlin, Germany) was spin-coated onto a 10 mm glass wafer at 845 rpm for 30 s to reach a height of 3 μm. The coated wafer was soft-baked at 95 °C for 2 min. Then, the wafer was covered with the first chrome mask (Delta mask, Enschede, Netherlands) and exposed to a collimated light source with a wavelength of 365 nm (model 30; OAI, San Jose, CA, United States) with a dose of 60 mJ/cm^2^. The first photomask was designed to fabricate 140-180 tapered microchannels with a width of 15 μm, decreasing towards 3 μm, and with a length of 450 μm (layout Editor CleWin version 3.0.11, Hengelo, Netherlands). Then the exposed wafer was post-baked at 95 °C for 2 min and developed with the SU-8 developer (micro resist technology GmbH). Prior to the second layer, the wafer was treated with oxygen plasma at 310–320 mTorr for 20 s and spin-coated with SU-8 50 (micro resist technology GmbH) at 2000 rpm for 30 s to reach a height of 50 μm. Next, the wafer was incubated for 30 min at room temperature and soft-baked by increasing the temperature to 65 °C at a rate of 1°C/min, followed by 6 min at 65 °C, and then increasing to 95°C at a rate of 1°C/min, and incubated for another 20 min at 95°C. The wafer was gradually cooled down to room temperature before being exposed to UV light with a dose of 200 mJ/cm^2^ through the second chrome photomask. This photomask was designed to fabricate the two main compartments of the chip. The wafer was post-baked by increasing the temperature to 65 °C with 1 °C/min, incubated for 1 min at 65 °C, increasing to 95°C with 1°C, and incubated for 5 min at 95°C. After cooling down to room temperature, the wafer was developed with the SU-8 developer, followed by a hard bake step for 20 min at 150°C. The master was incubated with 10 µL Trichloro (1H,1H,2H, 2H-perfluorooctyl)silane (Sigma Aldrich, Zwijndrecht, Netherlands) for 1 h in a desiccator to remove PDMS more easily from the SU-8 masters. A mixture of the PDMS-prepolymer and curing agent (10:1 weight ratio; Sylgard^®^184, Mavon B.V. Alphen aan de Rijn, Netherlands) was cast to the master to achieve a thickness of 3 mm. Then, PDMS was cured on a hotplate at 70 °C for 70 min. After curing, the PDMS slab was cut in shape, and 8 mm holes were made using a biopsy punch (Kai, D-care BV, Houten, Netherlands) to create reservoirs that hold culture media in the respective compartments (Peyrin et al., 2011).

To fabricate perfluoropolyether (PFPE) molds, the PDMS chip was bonded to a PDMS box (50 × 50 × 7 mm; LxWxH). A mixture of Fluorolink MD 700 (Acota Ltd., Shropshire, UK) and 2% photoinitiator 2-Hydroxy-2-methylpropiophenone (Sigma Aldrich, Zwijndrecht, Netherlands) was poured into the box and exposed to N_2_ for 20 min to remove oxygen (Vitale et al., 2012). The mixture was cured at 5–7 J/cm^2^ in a blue wave LED flood-curing system (Dymax Europe GmbH, Wiesbaden, Germany), depending on the amount of the mixture. Then the PFPE mold was removed from the PDMS chip (Jellali et al., 2016).

Each PFPE mold was filled with 1.5 g of PDMS mixture to create chips with a thickness of 2–3 mm. PDMS was cured in an oven at 70°C for 1.5 h. PDMS chips were removed from the molds using a spatula. Chips were cut, and a puncher was used to open the reservoirs if necessary. The PDMS chamber was sealed with a 24 × 50 mm coverslip (Menzel Gläser) using oxygen plasma at 310–320 mTorr for 20 s and filled with water immediately. The chips were sterilized by incubating with 70% ethanol for 5 min at room temperature and washed 3 times with UP water. The chips are kept in a Petridish with moist filter paper to avoid evaporation of fluids.

### Culturing and differentiating SH-SY5Y cells

Neuron-like SH-SY5Y cells were cultured in DMEM:F12 supplemented with 10% heat-inactivated FBS, l-glutamine (2 mM), penicillin (100 U/mL), and streptomycin (100 μg/ml) (Life Technologies, #15070-063). Once the cells grew confluent, the SH-SY5Y cells were passaged using trypsin. The cells were incubated at 5% CO_2_ and 37°C. The cells were tested frequently for the presence of *mycoplasma*. For differentiation, SH-SY5Y cells were cultured in DMEM (DMEM; Life Technologies, United Kingdom, #42340-025) containing 1% heat-inactivated FBS, l-glutamine (2 mM), penicillin (100 U/mL), and streptomycin (100 μg/ml), freshly supplemented with 10 μM all-trans retinoic acid (r.a. cat. R2625, Sigma-Aldrich).

### Culturing ASM cells

Immortalized human ASM were cultured in DMEM supplemented with 10% Fetal Bovine Serum (FBS), 2.2% Penicillin-Streptomycin, and 0.6% Amphotericin B. Once the cells grew confluent, the ASM cells were passaged using trypsin. The cells were incubated at 5% CO_2_ and 37 °C. The cells were tested frequently for the presence of *mycoplasma*.

### Statistical analysis

The data are presented as mean ± standard error of the mean (SEM). Statistical differences between distinct conditions were calculated using a two-way ANOVA or mixed effect analysis followed by either a Dunnett’s test or a Tukey’s multiple comparisons test to calculate significant differences comparing three or more variables. A paired *t*-test was performed comparing two variables. Statistical analyses were performed in GraphPad Prism (version 9.3.0), and performed tests are specified in the figure legends.

## Results

### Derivation of vagal NCCs from hPSCs

**FIGURE 1:**
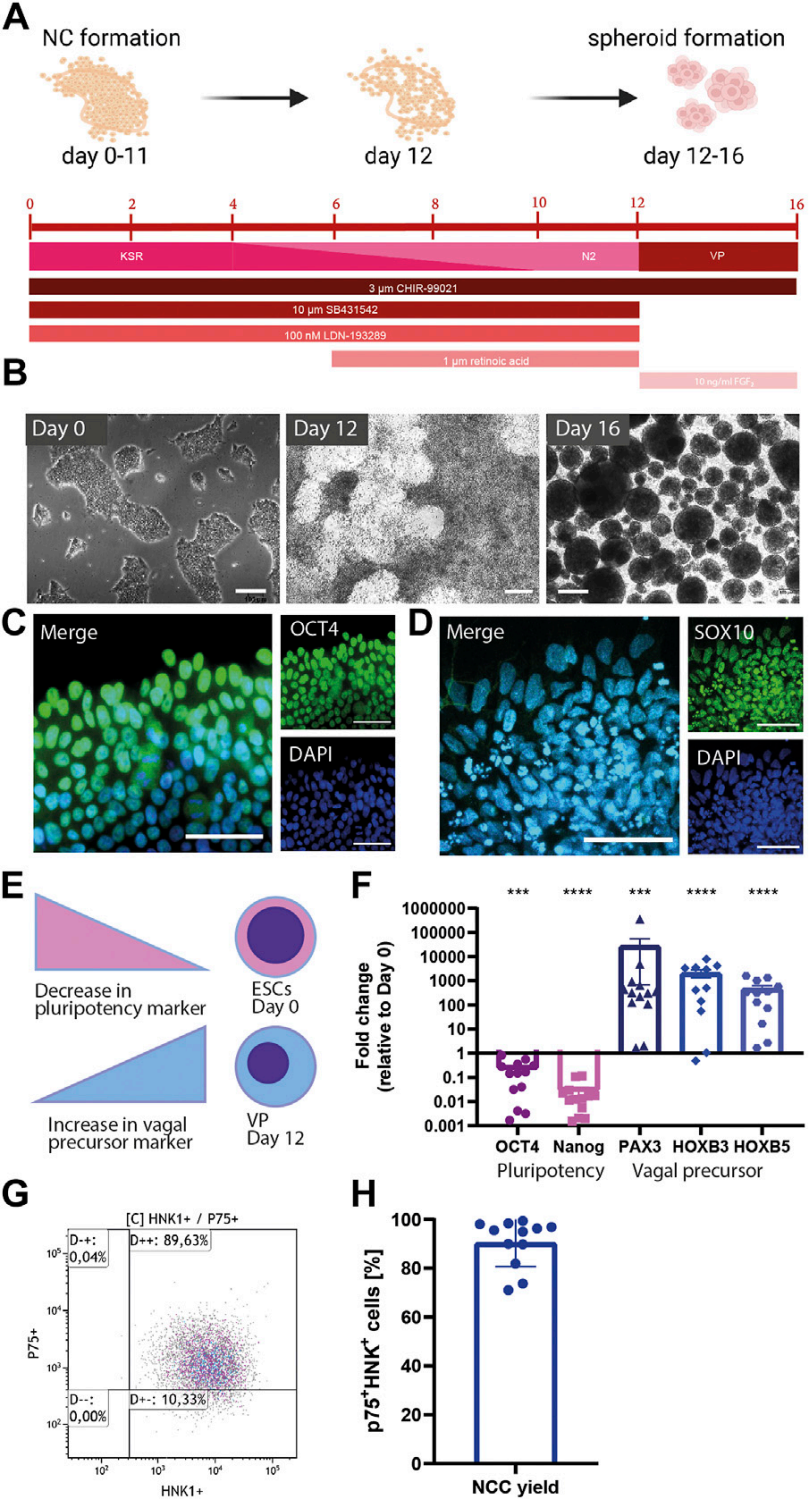
Derivation of vagal NCCs from hPSCs. **(A)** Schematic overview of vagal NCC induction from hPSCs. H9WA09 cells were directed into a vagal NCC faith using dual SMAD inhibition, and early WNT activation, r. a. Was added to direct NCCs from a cranial into a more distal phenotype. Knock-out serum replacer medium (KSR) was gradually replaced by N2 medium. Vagal NCCs were cultured in spheroids for 4 days to complete maturation. Created with BioRender.com. **(B)** Bright images of the differentiating cells over time. Scale bar = 200 µm. **(C)** OCT4 expression of Day 0 pluripotent cells. Scale bar = 50 µm. **(D)** SOX10 expression of Day 16 vagal NCC spheroids. Scale bar = 50 µm. **(E,F)** Gene expression of several markers for pluripotency, NCC, and vagal NCC. After 12 days, a decrease in the pluripotency genes OCT4 and Nanog was observed. The NCC marker PAX3 was upregulated, in combination with the vagal NCC markers HOXB3 and HOXB5 (N = 13) **(G,H).** FACS analysis showed that 90.48% (±2.83%) of the vagal NCCs were p75+-HNK1+ double positive. (N = 12) A paired *t*-test was performed to calculate statistical significance between means. ***p < 0.001; ****p < 0.0001 compared to day 0.

During embryonic development, airway cholinergic neurons originate from vagal NCCs. H9WA09 cells were directed into vagal NCCs using dual SMAD inhibition and early WNT activation using chemically defined conditions (Figure 1A) (Barber et al., 2019). To induce dual SMAD inhibition, SB431542 and LDN193189 were used as a BMP/TGF-β pathway inhibitor and a BMP pathway inhibitor, respectively. Later during the NCC differentiation, CHIR99021 was added for temporal WNT activation. A monolayer of pluripotent H9WA09 colonies differentiated into vagal NCCs in 12 days (Figure 1B). Following vagal NCC induction, cells were cultured as spheroids for 4 days in order to further mature the NCC phenotype (Figure 1B, right panel). On day 0, colonies were positive for OCT4, a marker for pluripotency (Figure 1C), while after 16 days of differentiation, cells showed decreased OCT4 expression and shifted to SOX10^+^ NCCs (Figure 1D). The NCC stem cell marker Nestin was detected in day 16 spheroids (Supplementary Figure S1A).

To confirm the vagal NCC identity of the acquired cells, we performed a gene expression analysis of NCC (*PAX3*) and vagal NCC (*HOXB3*, *HOXB5*) markers. Firstly, *PAX3* is reported to be one of the earliest markers of NCC induction (Monsoro-Burq, 2015). In addition, vagal NCCs express *HOXB3* and *HOXB5* during embryogenesis (Chan et al., 2005; Kam and Lui, 2015). After 12 days of differentiation, vagal NCCs displayed lower expression of pluripotency genes (*OCT4*, *p* < 0.001; *NANOG*, *p* < 0.0001) and higher expression of NCC genes (*PAX3*, *p* < 0.001; *HOXB3*, *p* < 0.0001; *HOXB5*, *p* < 0.0001) compared to day 0 (Figures 1E,F). On day 12, the normalized expression of OCT4 and NANOG was significantly downregulated compared to day 0 (Figure 1F). Comparison of these relative gene expressions between samples collected during differentiation revealed a population transition from pluripotent stem cells to vagal NCCs. *PAX3* is involved in NCC development; *PAX6*, on the other hand, is an important early marker for neural tube formation and subsequent central nervous system differentiation (Monsoro-Burq, 2015). The obtained NCCs showed a high expression of *PAX3*, whereas *PAX6* was completely absent (Supplementary Figure S1B).

The efficiency of the differentiation protocol was analyzed by determining the yield of NCCs using FACS. HNK1 and p75 are both surface markers that are highly abundant on the surface of migratory NCCs (Betters et al., 2010). Figure 1G shows a representative FACS analysis at day 12 of differentiation. Induction of H9WA09 cells towards a NCC fate was highly efficient, with 90.48% (±2.83%) of total cells being double positive for p75 and HNK1 (Figure 1H). Together, these data indicate that vagal NCCs (displaying HOXB3 and HOXB5) were induced with high efficiency.

### Neuronal differentiation from vagal NCCs

**FIGURE 2:**
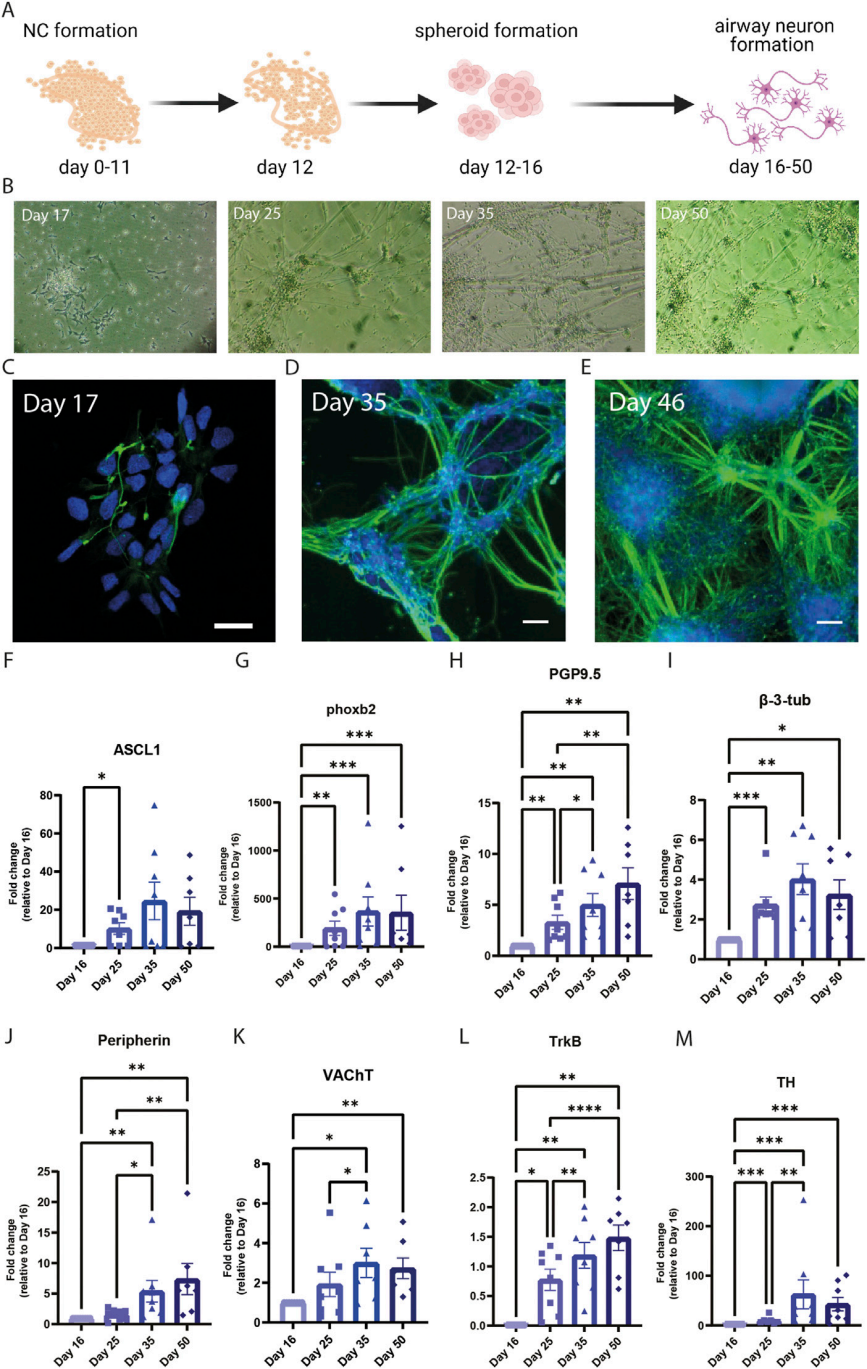
Differentiation of peripheral cholinergic neurons. **(A)** Schematic overview of hPSC differentiation into peripheral cholinergic neurons. Created with BioRender.com. **(B)** Brightfield images of the differentiating cells over time. **(C–E)** Immunofluorescence images of differentiating cells over time, showing the development of β-3-tubulin expression. Over time a greater neuronal network is being formed. Day 17, scale bar = 20 µm. Day 35 and day 46, scale bar = 100 µm. **(F–L)** Gene expression of airway cholinergic neuronal development over time. Gene expression was examined at different time points: Day 16, day 25, day 35, and day 50. ASCL1 **(F)** and phoxb2 **(G)** are essential for neuronal development and increased over the course of day 50. The pan-neuronal markers PGP9.5 **(H)** and β-3-tubulin **(I)** indicate the formation of neurons, in combination with peripherin **(J)** they indicate the development of peripheral neurons. VAChT **(K)**, indicating the formation of cholinergic neurons, was higher expressed over the course of 50 days. TH **(L)** showed a peak at day 35 before declining in expression towards day 50. TrkB **(M)** increased in expression over the course of 50 days (N = 8) A mixed-effect analysis followed by Tukey’s multiple comparisons test was performed to calculate statistical significance between means. *p < 0.05; **p < 0.01; ***p < 0.001; ****p < 0.0001, compared to day 16.

To differentiate vagal NCC precursors further into mature peripheral cholinergic neurons suitable for studies in to airway neuronal plasticity, we plated day-16-spheroids on FB/LM-coated surfaces and used BDNF as a main neurotrophic factor in the media in view of its prominent role in guiding neuronal development in the lung *in utero* (Figure 2A). A neuronal network was formed gradually and increased every day, with the first axonal outgrowth already visible after 24 h (Figure 2B, left panel; Figure 2C). At day 25 of differentiation, a neuronal network was clearly visible, which further expanded and became denser over time. β-3-tubulin staining confirmed the overall neuronal network formation (Figures 2C–E).

To track the development of cholinergic neurons during differentiation, we compared the gene expression between samples collected at several time points during the differentiation protocol. *In utero*, vagal NCCs express PHOX2B and ASCL1 after their inclusion in the foregut (Hao and Young, 2009). Accordingly, differentiated cells showed a relative increase in gene expression of *PHOX2B*, and *ASCL1*, with a peak at day 35 for *ASCL1* (Figures 2F,G). The expression of both the *TUBB3* and *UCHL1* confirmed that neurons were abundantly present and that their marker gene expression increased from day 16 to day 50 (Figures 2H,I). Expression of peripherin (*PRPH*), a cytoskeletal protein found in peripheral neurons, also increased towards day 50 and confirmed a peripheral neuronal phenotype (Figure 2J). Vesicular acetylcholine transporter (VAChT, *SLC18A3*) expression confirmed the cholinergic phenotype of the neurons (Figure 2K).

Both VAChT (*SLC18A3*) and TrkB (*NTRK2*) increased over the course of 50 days, indicating neuronal formation and maturation (Figures 2K–M). Tyrosine hydroxylase (*TH*) is a marker for sympathetic neurons; however, it is also a precursor for cholinergic neurons during development (Weihe et al., 2006). Consistent with this contention, *TH* peaked in expression at day 35 of differentiation and declined afterward (Figure 2L). Our data implies that the cells matured over time into peripheral cholinergic neurons as *TH* expression decreased (Figure 2L), and the expression of *PRPH* and *SLC18A3* increased.

**FIGURE 3:**
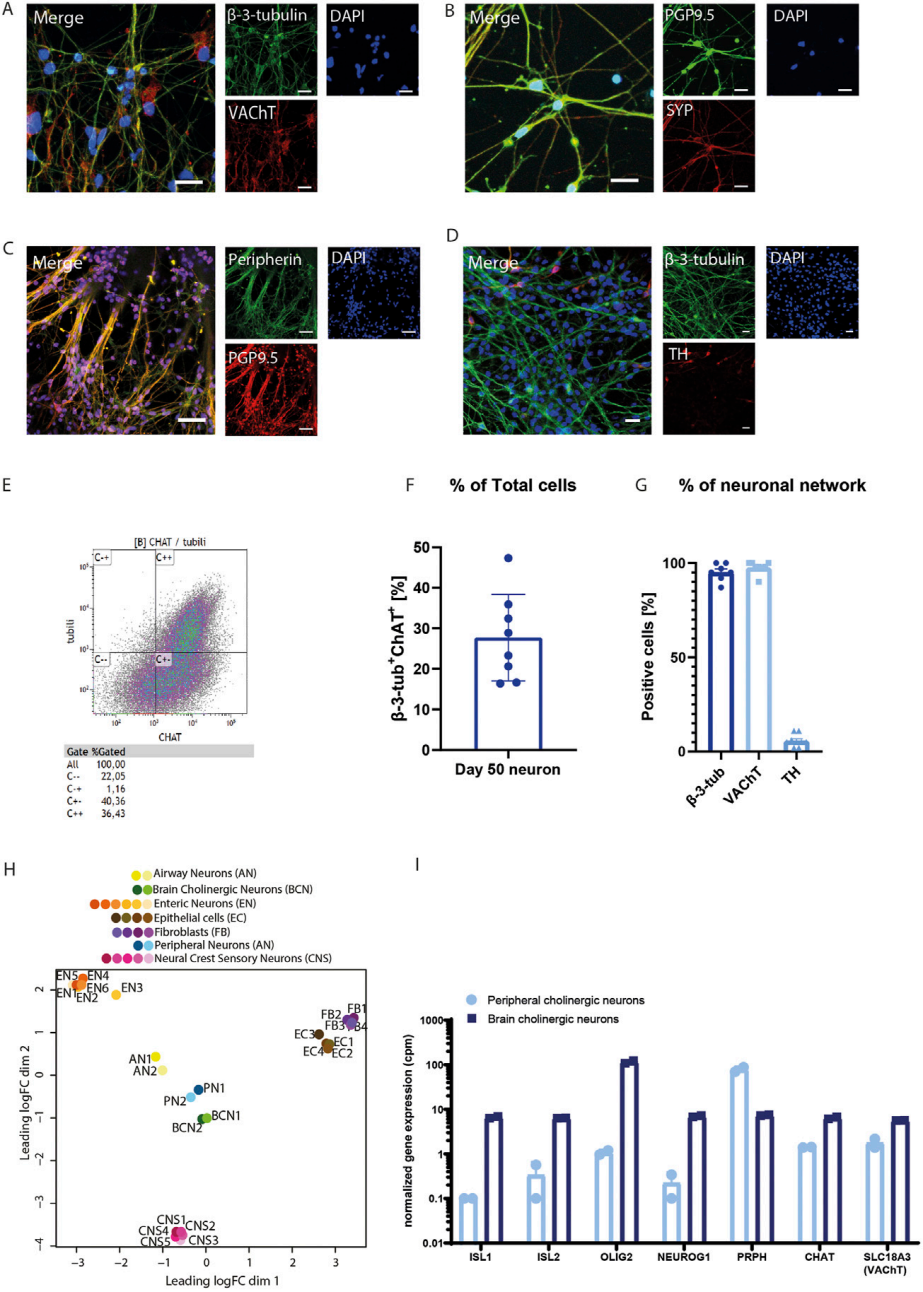
Peripheral cholinergic neurons **(A–D)**. Immunofluorescence images characterizing neurons. β-3-tubulin or PGP9.5 were used as a pan-neuronal marker. The neurons show the presence of VAChT **(A)** after 50 days of differentiation, in addition to SYP **(B)** and peripherin **(C)**. A smaller proportion of the neurons show TH^+^ after 50 days. **(E–G)** FACS analysis demonstrated that 27.7% (±3.8%) of the neurons are β-3-tubulin^+^-ChAT^+^, indicative of cholinergic neurons. A representative FACS analysis plot is shown in E, panel F shows the quantification (n = 8). **(G)** Quantification of IF staining of β-3-tubulin (n = 6), VAChT (n = 8), and TH (n = 8) showed that the majority of sprouting cells expressed β-3-tubulin and VAChT, whilst TH was only sparsely present. **(H)** PCA analysis of RNAseq data showed that hPSC-derived airway neurons were distinct from airway epithelial cells or fibroblasts, but showed more overlap with hPSC-derived peripheral neurons or brain cholinergic neurons. **(I)** Despite similarities with brain cholinergic neurons in the PCA analysis, peripheral cholinergic neurons showed a distinct expression of central and peripheral markers with the central markers ISL1, ISL2, OLIG2 and NEUROG1 being enriched in brain cholinergic neurons and the peripheral marker PRPH being enriched in peripheral cholinergic neurons. Figures were presented as mean ± SEM.

Further identification of neuronal identity revealed that after 50 days, most of the generated airway neurons were VAChT^+^ (Figure 3A), indicating a cholinergic phenotype. Neurons matured gradually into airway cholinergic neurons after 35 days of differentiation. On day 35, neurons stained positive for β-3-tubulin, while VAChT expression was sparse (Supplementary Figure S2A). Other important markers of the neuronal network include the presence of synaptophysin (SYP) and peripherin (Figures 3B,C). We observed TH expression using IF staining, but found that only a minor proportion of the generated neurons was TH^+^ (Figures 3D,G), in contrast to abundant VAChT expression (Figures 3A,E–G). TH^+^ neurons were not overlapping with VAChT^+^ neurons (Supplementary Figure S2B). Most of the neurons appeared Peripherin^+^ at day 50, indicating that the obtained neurons are PNS neurons, not central nervous system neurons (Figure 3C). Also, part of the neurons expressed SYP, indicating the presence of synapses. In autonomic neurons, synapses are distributed over the length of the axons, rather than having a synapse formed at the extremity end of the axons, which was also observed here (Figure 3B).

Airway cholinergic neurons use ACh as their primary neurotransmitter, which is produced by the presence of the ACh synthesizing enzyme ChAT. While direct assessment of ACh release by these cells is not possible due to insufficient sensitivity of such assays, FACS analysis further confirmed the final cholinergic phenotype of day 50 of neuronal differentiation and maturation. After 50 days of differentiation, 27.6% (SEM ±4.5%) of the totalcell population was β-3-tubulin^+^-ChAT^+^ (Figures 3E–G). Of importance, this is a percentage of total cells, including cells from the vagal spheroid as well as the cells that sprouted from the spheroids into neuronal networks. When the proportion of β-3-tubulin^+^ cells was analyzed for ChAT expression, 80.0% (SEM ±6.5%) were ChAT^+^ cells. Zooming in on the sprouted neuronal network only (excluding the vagal spheroids), quantification of the IF staining of these cells showed that 95.1% (SEM ±4.6%) of the sprouted cells were β-3-tubulin^+^ and 97.6% (SEM ±3.3%) of the cells were VAChT^+^ (Figure 3G), supporting the conclusion that the majority of the neurons sprouted from the vagal spheroids are cholinergic.

RNA sequencing analysis of two samples at day 50 of differentiation provided further support of the identity of the peripheral cholinergic neurons. Neuronal markers were abundantly present and in higher numbers than other neural crest derivatives like glial cells, mesenchymal cells, melanocytes, or endocrine cells. The pan-neuronal markers *NEFM* and *UCHL1* showed the highest read counts compared to other cell types (Supplementary Figures S3A-C). Mature peripheral cholinergic neurons display the autonomic ganglia-specific nicotinic and muscarinic ACh receptors: *CHRNA3*, *CHRNB4*, *CHRM2*, *CHRM3*, and *CHRM4* (Coulson and Fryer, 2003; Hollenhorst and Krasteva-Christ, 2021). These ACh receptors, especially the nicotinic receptor 3 (*CHRNA3*) and muscarinic receptor 3 (*CHRM3*), were present in the generated neurons. In addition, the glutamate receptor *VGLUT2* was abundantly expressed on day 50 neuronal differentiation, as well as the vesicular monoamine transporter *VMAT2I* (Supplementary Table S3).

We performed a comparison to determine the resemblance of the generated neurons with other cells types through principal component analysis (PCA). PCA is a statistical technique to summarize the information from extensive databases to improve interpretability while keeping as much information as possible (Jolliffe and Cadima, 2016). We compared the RNAseq profile of the obtained neurons with previously published datasets using hPSC-derived peripheral neurons (Lyoo et al., 2022), hPSC-derived neural crest sensory neurons (Nickolls et al., 2020), human-induced brain cholinergic neurons (from fetal fibroblasts) (Liu et al., 2013), human enteric neurons (May-Zhang et al., 2021), human epithelial cells, and human fibroblasts. Interestingly, the gene expression pattern from generated neurons most closely resembles that of hPSC-derived peripheral neurons and brain cholinergic neurons. On the other hand, enteric neurons, fibroblasts, epithelial cells, and neural crest sensory neurons remained quite distinct from the generated airway neurons with the current protocol (Figure 3H). While the peripheral cholinergic neurons showed expected similarities in gene expression to CNS cholinergic neurons, important differences exist between these subtypes that mark their different developmental origins. Crucially, the peripheral cholinergic neurons were negative in expression or showed limited expression of the neural tube markers *ISL1*, *ISL2*, *OLIG2* and *NEUROG2*, which were expressed in abundance by CNS cholinergic neurons (Figure 3I). In contrast, the peripheral cholinergic neurons were enriched in *PRPH* and in the vagal marker PAX3 (Figure 3I and Supplementary Figure S1).

Next, we investigated whether the generated neurons were functional using a multi-electrode array (MEA). Recordings of a MEA enable the measurement of neurons’ spontaneous firing to validate neuronal maturation. Over the course of day 25 through day 73, neurons became more spontaneously active (Supplementary Figure S4).

### Generation of a two-compartment microfluidic chip allowing directional axonal outgrowth and separate cell culture

PNS neurons extend their axons into peripheral tissues, and communicate with a diversity of target cells. Therefore, we designed and fabricated an *in vitro* cell culture device that mimics axonal communication (Taylor et al., 2005; Peyrin et al., 2011). Using PDMS, we fabricated a culture chamber with two separate compartments connected by microchannels, as outlined in Supplementary Figure S5 and Supplementary Figure S6.

The device ensured a separate culture of different cell types in their culture medium: hydrostatic pressure prevented the mixing between the media from the somal and the axonal compartment. (Supplementary Video S2, demonstrated with food coloring).

A microfluidic neuron culture chip design is commercially available (Xona Microfluidics^®^) and consists of two main compartments, connected by an array of 3 μm by 3 μm microchannels that allow axonal communication between these chambers without mixing Soma (Supplementary Figure S6B, left panel). In initial experiments using SH-SY5Y cells, however, the channels were found too small for our purpose, and we could not guide axons into the axonal compartment. Altering the design to a bigger microchannel of 10 μm by 10 μm (Supplementary Figure S6B, center panel) resulted in axonal outgrowth; however, it also resulted in somal bodies moving into the axonal compartment. With this knowledge, we next developed a third design, inspired by Peyrin et al., which encompassed arrays of rectangular microchannels of decreasing width (Supplementary Figure S6B, right panel) (Peyrin et al., 2011). The resulting microfluidic chip also comprised two distinct cell culture chambers as the somal and axonal compartments. The compartments were connected by a series of 450 μm long, 3 μm high, asymmetrical microchannels starting at 15 μm entrance allowing optimal neuronal collection, followed by the narrow 3 μm tapering of the microchannels to promote unidirectional growth of axons and to ensure the confinement of SH-SY5Y cell bodies to the somal compartment (Supplementary Figure S6A).

### hPSC-derived cholinergic neuron differentiation and characterization on a chip

**FIGURE 4:**
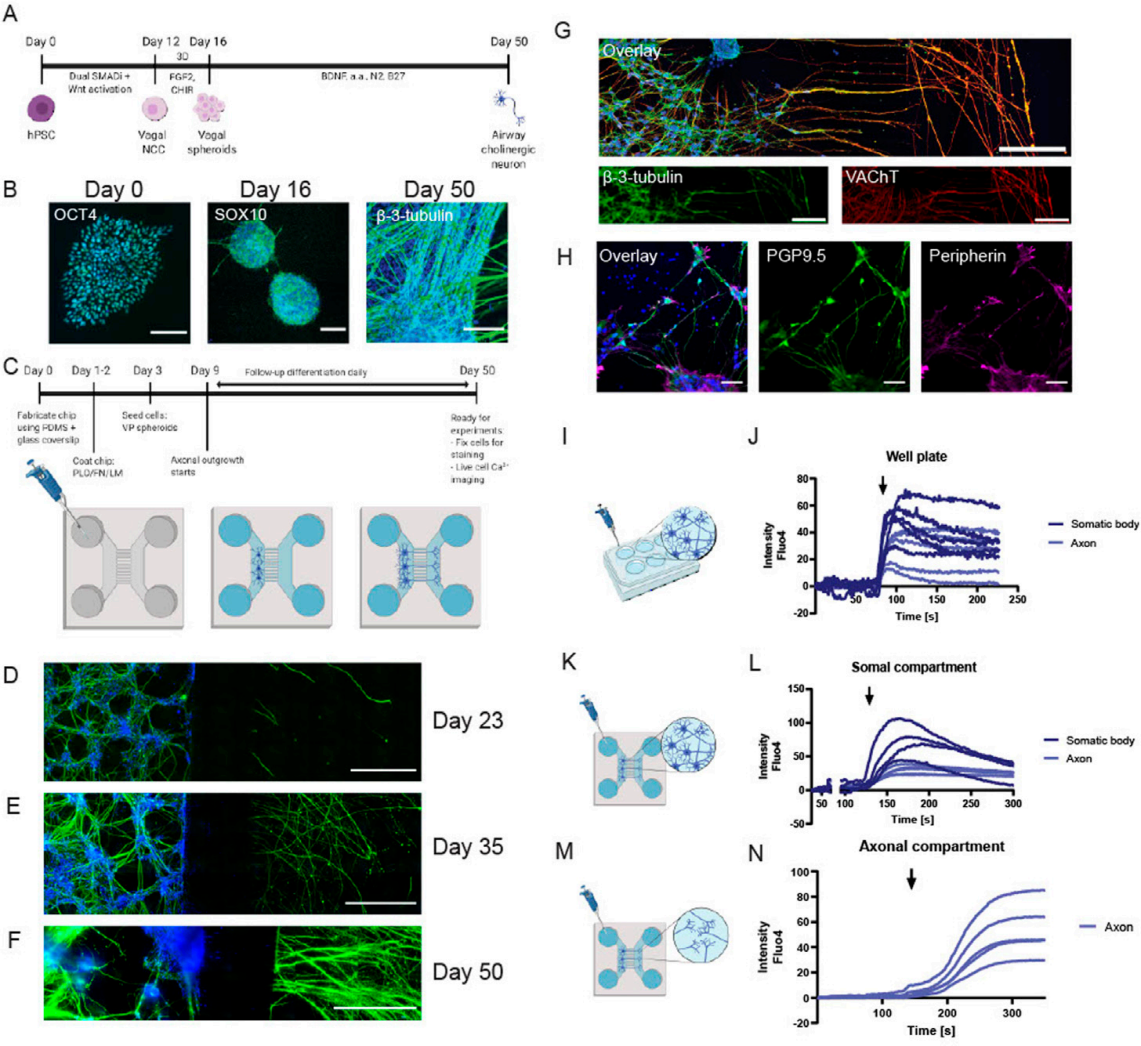
hPSC-derived peripheral cholinergic neurons on a chip. **(A)** Timeline of differentiation of peripheral neurons from hPSCs via vagal NCCs. In 50 days, peripheral cholinergic neurons were generated in the presence of BDNF. **(B)** If images of different stages of differentiation of hPSCs. Left: OCT4^+^ hPSC colony at day 0 of differentiation. Scalebar = 200 µm. Middle: SOX10^+^ vagal NCC spheroids at day 16 of differentiation. Scalebar = 200 µm. Right: β-3-tubulin^+^ airway neurons at day 50 of differentiation. Scalebar = 50 µm. **(C)** Schematic overview of the timeline of stemcell seeding into the chips after fabrication; the chips were first coated with PLO/LM/FB, after which vagal NCC spheroids were dissociated and seeded into the chip. The medium was changed 2-3 times per week. Axons started to grow out of the microchannels into the axonal compartment after 6 days. Neurons were cultured into the chip up to day 50. **(D–F)**: IF images of β-3-tubilin^+^ neurons showing increased axonal outgrowth over time. Scalebar = 500 µm **(D)**. At day 23 of differentiation (7 days on chip) some axonal outgrowth was visible, which increased towards day 35 **(E)** and day 50 **(F)**. **(G,H)** IF images of peripheral neurons showing the majority of neurons was VACHT^+^ (G, scalebar = 200 µm) and peripherin^+^ (H, scalebar = 50 µm). **(I–N)**. Schematic overview of Ca^2+^ imaging of hPSC-derived neurons cultured in six wells-plates or on-chip, plus traces of Ca^2+^ response of the generated neurons. KCl (60 mM) was added to the neurons or to a medium reservoir of the somal compartment. The time point of adding KCl is indicated with a black arrow. I-J. KCl stimulation and Ca^2+^ response of generated neurons cultured in a six wells-plate. **(K–L)** KCl stimulation and Ca^2+^ response of generated neurons cultured on-chip, neurons and axons were imaged in the somal compartment. **(M,N)** KCl stimulation and Ca^2+^ response of generated neurons cultured on-chip, axons were imaged in the axonal compartment. Shown are five representative traces for each experimental design, taken from n = 5 differentiations, each performed in 1-3 separate chips. Created with BioRender.com.

Airway neurons were differentiated from hPSCs following the above described protocol (Figures 4A,B). The vagal NCCs were derived in six wells-plates in 16 days, and VP spheroids were plated into PLO/LM/FB coated chips for the remainder of the 50-day-differentiation period (Figure 4C). After 6 days of on-chip differentiation (day 22 of the hPSC-differentiation protocol), axons typically start to extend into the axonal compartment (Figures 4C,D). Over time, as the network of airway neurons expands, the extension of axons into the axonal compartment increases tremendously (Figures 4E,F). On-chip staining of hPSC-derived airway neurons confirmed the presence of vesicular acetylcholine transporter (VAChT), an important marker for airway cholinergic neurons (Figure 4G). In addition, the airway cholinergic neurons are peripherin^+^ (Figure 4H). A Ca^2+^ response to KCl in live cells was used to demonstrate the functionality of hPSC-derived airway cholinergic neurons. Imaging using conventional culture was compared to on-chip imaging of the generated neurons directly in the chip (Figures 6I–N). Using direct on-chip imaging, one can choose to image either the mix of somal bodies and axons in the somal compartment, or to visualize only axons in the axonal compartment. KCl was always added directly onto the somal bodies or into the somal compartment, where KCl stimulated the cells within seconds. Traces of Ca^2+^ were followed using FLUO-4 AM and quantified (Figures 4J,L,N). Imaging of somal bodies of cells cultured in a six wells-plate (Figures 4I,J, Supplementary Video S3) showed a similar Ca^2+^ response to KCl compared to somal bodies cultured on-chip and imaged in the somal compartment (Figures 4K–L, Supplementary Video S4). The Ca^2+^ response of axons measured in the axonal compartment was clearly present, even though cells were stimulated in the somal compartment. The Ca^2+^ response extended all the way into the axonal compartment (Figures 4M,N, Supplementary Video S5).

## The axon-guidance chip is biocompatible and does not differ from conventional cell culture systems

**FIGURE 5:**
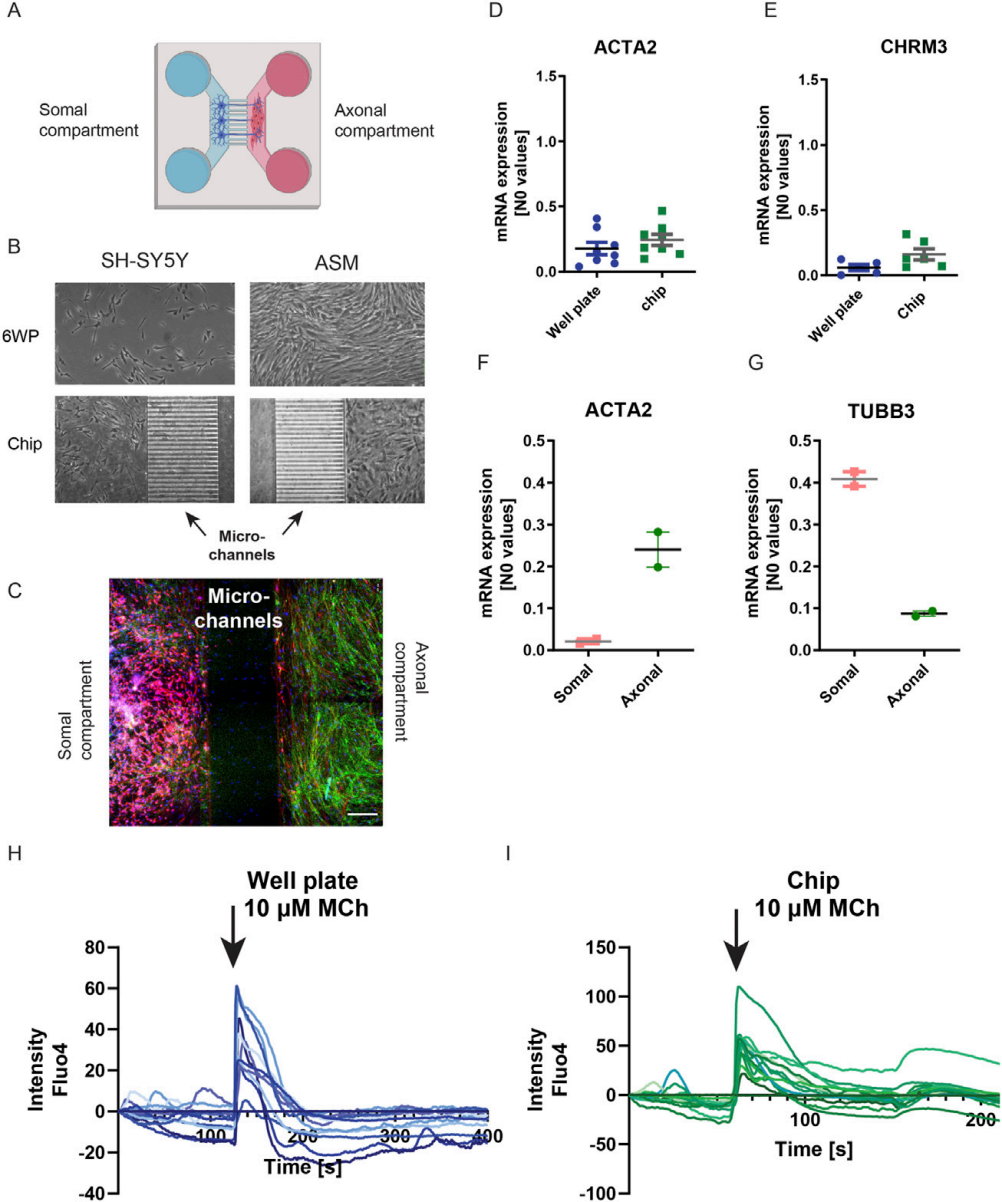
Comparing cell culture on-chip and in conventional cell culture. **(A)** Schematic overview of co-culture of neurons and ASM cells in the chip. In the left main compartment (blue) of the chip, neurons were seeded and in the right main compartment (red) ASM cells were seeded. Axons connected the two main compartments by growing for the neuronal to the axonal compartment. To compare, cells were cultured in a 6WP. Created with BioRender.com. **(B)** Left Brightfield images of neuronal-like SH-SYSY cells cultured both in a 6WP and in a chip. Right: Brightfield images of ASM cells cultured both in a 6WP and in a chip. Both SH-SY5Y and ASM cells had a similar morphology independent of the culture vessel. **(C)** IF image of an ASM-neuronal co-culture on-chip showed that both cell types are confined to their own compartment. Red: β-3-tubulin; green: Phalloidin. Scalebar = 200 µm. **(D,E)** mRNA expression of ASM cells cultured both in the chip and in a 6WP. The expression of ACTA2 and the CHRM3 receptor are similar for both culture vessels. F-G mRNA expression of cells isolated from individual channels. **(F)** ACTA2 expression of cells from either the somal compartment or the axonal compartment showed that ACTA2 was mainly expressed in the axonal compartment. **(G)** TUBB3 expression of cells isolated from either the somal or the axonal compartment showed that TUBB3 was mainly expressed in the somal compartment. **(H,I)** Live cell Ca^2+^ imaging using Fluo-4. MCh (10 µM) stimulation of ASM showed a similar trace for ASM both cultured in a 6WP or on-chip. The time point of adding MCh is indicated with a black arrow. Shown are individual traces of 10 regions of interest taken from n = 6 chips.

ASM bundles in the lung are densely innervated, making ASM cells an interesting cell type to implement in co-cultures with neurons. To implement two co-culturing capabilities of the chip, neuronal-like SH-SY5Y cells and ASM cells were initially cultured in the chip simultaneously, in the somal and axonal compartment, respectively (Figure 5A). We investigated if an on-chip culture of cells would compromise their morphology and functionality compared to culture in plastic 6-well plates (6WP) and explored the advantages of on-chip culture. First, cell morphology did not differ between on-chip culture and 6WP culture for both the SH-SY5Y cells and the ASM cells (Figure 5B). The SH-SY5Y cells did not extend long neurites into the axonal compartment, as their neurites remain relatively short after differentiation. Still, the SH-SY5Y cells proved a suitable cell type to validate and optimize chip characteristics. An advantage of mounting the PDMS chip to a microscopic glass coverslip is that the coverslip allows on-chip staining and imaging. On-chip IF staining showed the confinement of both cells to their compartment (Figure 5C).

Comparing gene expression of the smooth muscle markers ACTA2 (α-sm-actin) and CHRM3 (M_3_ receptor) of ASM cells both on-chip and in 6WPs showed no difference in expression between culture vessels (Figures 5D,E). Comparing ACTA2 expression of cells from either the somal compartment or the axonal compartment showed that ACTA2 mRNA was mainly expressed in the axonal compartment, in which ASM cells were cultured (Figure 5F). Similarly, the neuronal marker TUBB3 (β-3-tubulin) was mainly expressed in the somal compartment when comparing its gene expression of mRNA isolated from either the somal or the axonal compartment (Figure 5G).

Using the glass coverslip, live-cell fluorescent imaging using FLUO-4 AM could be performed directly on-chip as well. Stimulating ASM cells with 10 µM of methacholine (MCh), a muscarinic receptor agonist, showed similar responses in ASM cultured on coverslips on a 6WP compared to on-chip culture (Figures 5H,I). Collectively, this indicates that the chips can harbor and grow cells preserving phenotypic and functional features compared to conventional methods, with the advantage of direct on-chip imaging techniques and mRNA isolation of individual cell types after co-culture.

**FIGURE 6:**
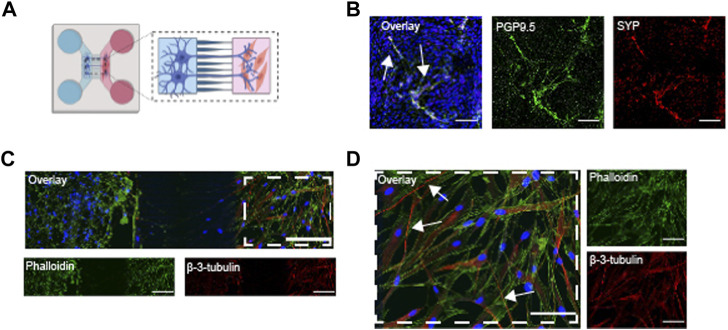
Co-cultures of hPSC-derived airway neurons and ASM cells on a chip **(A)** Schematic overview of the stem cell and ASM cell seeding into the chips after fabrication; Vagal NCCs were seeded and cultured in PLO/LM/FB coated chips up to day 45. The medium was changed 2-3 times per week. At day 45, ASM cells were seeded in the axonal compartment. Both cells were co-cultured for 5 days, up to day 50. **(B)** IF image of co-culture showing the expression of PGP9.5 and SYP in generated neurons. Scalebar = 50 µm. **(C)** IF image ASM cells and hPSC derived neurons. Phalloidin captured the actin-rich ASM cells and also the neurons (green). β-3-tubilin^+^ neurons innervate ASM in the axonal compartment (red). Scalebar = 200 µm. **(D)** Close-up of IF image of co-culture. White arrows indicate some innervating neurons (red). Scalebar = 100 µm. Created with BioRender.com.

Next, ASM cells were added to the chips together with the hPSC-derived airway cholinergic neurons to complement the device for neuro-effector interactions. Airway neurons were first differentiated in the somal compartment for 2 weeks. After confirming that axon bundles passed through the microchannels on day 45, ASM cells were added to the axonal compartment (Figure 6A). The chip allows the addition of cell culture media optimal for each cell type; thus, both cells were cultured in their respective culture media as mentioned in the materials and methods. After co-culturing the generated neurons with ASM cells, we found the expression of synaptophysin (SYP), a marker for presynaptic vesicles (Figure 6B), after co-culturing the generated neurons with ASM. Indeed, the neuronal bundles innervated the ASM cells in the axonal compartment (Figures 6B–D). Staining of β-3-tubulin revealed that axons integrated with and innervated the ASM cells (Figure 6D, white arrows).

## Discussion

Here, we established a cell culture protocol for the differentiation of peripheral cholinergic neurons suitable for studies on airway neuroplasticity along with a compartmentalized microfluidic device for the study of neuro-effector interactions in the lungs. We demonstrate the differentiation of peripheral cholinergic neurons *via* a vagal NCC precursor. The cholinergic phenotype, mainly present in the airways, was confirmed in our cultures by ChAT and VAChT expression. We improved the translational value of the model by creating an organ-on-chip model suitable for studying neuro-effector interactions. A PDMS chip was fabricated and successfully implemented. The PNS-on-chip model could serve as a useful experimental platform for monitoring neuro-effector interactions in various cell types and for investigating the effectiveness or safety of drugs.

The induction of the vagal precursors was highly efficient, with a >90% yield of HNK^+^-p75^+^ cells. This high efficiency is in accordance with vagal NCC induction for enteric neuronal development, showing a similar yield for NCC induction (Barber et al., 2019). The subsequent differentiation of vagal NCC into peripheral cholinergic neurons showed the formation of a neuronal network that kept increasing gradually, reflected both in protein and in gene expression. Protein expression analysis revealed that most neurons displayed a cholinergic phenotype. IF staining showed the utmost part of neurons to be VAChT^+^, while only a minor part was TH^+^. In addition, FACS analysis revealed ChAT expression in the majority of β-3-tubulin^+^ cells. Comparing the generated neurons with other cells using a PCA affirmed that the characterization mainly overlaps with peripheral and brain cholinergic neuronal samples. Although the cholinergic phenotype is similar to central nervous system (CNS) cholinergic neurons, as expected, the developmental origin of these neuronal subtypes is completely distinct. CNS neurons are derived from the neural tube, not the neural crest and in line with this, the peripheral cholinergic neurons were derived from an essentially pure vagal precursor population at day 12, positive for PAX3 and negative for the neural tube marker PAX6. The enrichment of *PRPH* and lack of/limited expression of the CNS markers *ISL1*, *ISL2*, *OLIG2* and *NEUROG1* confirms this peripheral neuronal identity and clearly distinguishes these neurons from CNS cholinergic neurons. Similarly, RNAseq analysis showed that neurons were the predominant cell type in our samples. VAChT expression confirms maturation of the cholinergic neurons over time: at day 35, VAChT is hardly present, while at day 50, VAChT is present in the majority of neurons.

Following the changes in gene expression, the neuronal differentiation follows a developmental maturation as observed *in vivo*. TH is considered a marker for sympathetic neurons. However, it is also a precursor for airway cholinergic neurons during development (Weihe et al., 2006). TH is expressed transiently during development: in neurons and neuroendocrine cells, TH is expressed in cells that in adulthood no longer express TH or only at very low levels (Weihe et al., 2006). The peak expression of *TH* observed at day 35 of differentiation could be explained by a transient TH expression. The obtained neurons still express *TH* after 50 days of differentiation, directing towards an incomplete neuronal maturation. However, IF staining showed a minor proportion of cells were TH^+^, which could be the transient expression or a small population of sympathetic neurons. In addition, gene expression for sensory neuronal markers was detected, indicating that although the cholinergic nerve population is highly enriched, minor populations of other neurons are present as well.

In the development of the chip, we passed through several optimization steps. The designed device was based on an improved version of the commercially available XonaChip, incorporating a funnel-shaped structure of the microchannels to improve axonal in- and outgrowth. Moreover, the microchannel dimensions (3 μm × 3 μm at the exit of the channels) ensure that cell bodies cannot travel from the somal compartment into the axonal compartment or *vice versa*.

The design of the chip incorporates several advantages over conventional cell culture techniques and other versions of microfluidic chips. First, microfluidic chips use less medium compared to conventional culture plates. In the case of hPSC culture, medium can be costly. A range of analyses, such as staining or gene expression analysis, was simplified or improved using the chip. In the current design, we could perform an IF staining on-chip directly: instead of making the chip completely out of PDMS, we added a glass coverslip at the bottom of the system, allowing on-chip imaging. Direct on-chip imaging makes cell characterization using IF staining or live-cell imaging to test functionality a very accessible process. In addition, this chip design is easy implementable in other labs. Many chips rely on the use of flow pumps. Flow rate pumps have the disadvantage of taking up much space in cell culture incubators (Perdigones, 2021). Such static chips can easily be cultured with many at a time: a significant advantage to scaling experiments up. The design was based on hydrostatic pressure to seed cells and to supply medium and nutrients (Taylor et al., 2005). Some cell types, like endothelial cells, rely on flow for proper differentiation (Kim et al., 2013). Neurons, on the other hand, thrive in static conditions for their differentiation (Wang et al., 2017). In our experiments, the system without flow pumps was not only convenient for culture but also encompassed better experimental outcomes.

As we observed, many of the seeded vagal NCCs in the chip differentiated into neurons with long neurites. The axons extended through the 450 μm long microchannels and formed dense bundles in the axonal compartment, allowing to learn more about the axonal communication between the cholinergic nervous system and the lungs. The bundles stained positive for VAChT and peripherin, confirming the formation of cholinergic neurons from the PNS, which are predominant in the lungs. Here, the neurite bundles gave a clear view of Ca^2+^ traces after KCl stimulation of the somal bodies independently. The outgrowth of bundles into the neurite compartment could already be used as a platform to understand neuronal outgrowth and development and to measure the response of axons after neuronal stimulation adequately. Many drugs have been developed in the central nervous system that target neuroplasticity, whereas drugs targeting the PNS are lacking (Goldsteen et al., 2020). Visualizing the neuronal network aids in developing PNS neuroplasticity models and subsequent drug development and testing.

A next challenge for an on-chip *in vitro* model would be this indirect activation of effector cells. Innervation of the airways during development relies on guidance from ASM (Aven and Ai, 2013), making ASM a logical cell type to implement in co-cultures with neurons. To implement patient-specific neuro-effector cultures, one could also differentiate smooth muscle cells from hPSCs. However, among the many differentiation protocols that are available for smooth muscle cells, none is able to generate tissue-specific smooth muscle cells (Goldsteen et al., 2021). We valued the inherent properties that ASM cells have to aid the differentiation into airway neurons specifically, hence our choice for immortalized human ASM cells. Still, now that the first step for an airway nervous system on a chip is established, other cell types can be used to co-culture with airway cholinergic neurons. Nevertheless, for each chosen effector cell used for the on-chip neuro-effector interactions applies: especially the individual mRNA isolation from each cell type might, for the first time, enable *in vitro* experiments to determine the contribution of each cell type to the onset of neuroplasticity.

The use of stem cell-derived sources of differentiated human cells has its advantages as these primary cells are often difficult to obtain. However, important limitations to this approach need to be acknowledged and our work is no exception. hPSC derived cells often maintain a relatively immature phenotype in comparison to primary cells. When comparing electrophysiological properties of hPSC-derived neurons and primary neurons using MEA recordings, our hPSC-derived neurons were found to have relatively limited spontaneous firing activity. Furthermore, while co-cultures in the chip can be established, it remains to be confirmed that such co-cultures adequately recapitulate the neuro-effector junction *in situ*. These are challenges on the horizon that are worthwhile resolving. Until that time, the hPSC derived neurons are likely most suitable for neurodevelopmental, neuronal differentiation and neuroplasticity related questions.

## Conclusion

In conclusion, we have demonstrated the ability to differentiate hPSCs into peripheral cholinergic neurons suitable for studies on airway neuroplasticity in asthma *via* a vagal precursor using chemically defined media. We have implemented these neurons on a two-compartment axon guidance chip for the study of airway neurobiology and established that phenotypic and functional characteristics of the cells are preserved in the microfluidic chip. The organ-on-chip device can provide a valuable tool as a human *in vitro* model that aids in the discovery of important neuro-effector mechanisms. In addition, understanding the pathophysiological mechanism of airway neuronal plasticity and the interaction of airway cholinergic neurons with effector cells might contribute to our understanding of neuro-effector interactions in asthma.

## Data Availability

The datasets presented in this study can be found in online repositories. The names of the repository/repositories and accession number(s) can be found below: https://www.ncbi.nlm.nih.gov/geo; GSE211478.
