# Supplementary material for: Differentiation and on axon-guidance chip culture of human pluripotent stem cell-derived peripheral cholinergic neurons for airway neurobiology studies
Source: Front Pharmacol. 2022 Oct 28;13:991072. doi: 10.3389/fphar.2022.991072 (PMC9651921; doi:10.3389/fphar.2022.991072)
Supplement: Supplementary file 3 [file DataSheet2.docx]

# Supplementary material


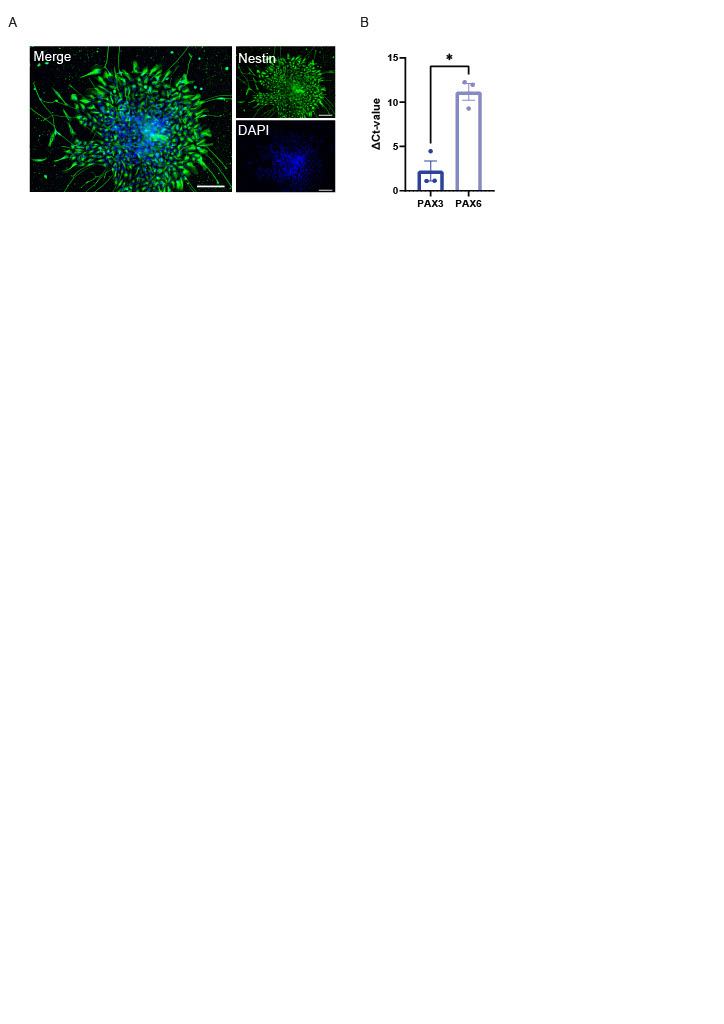


*Supplementary Figure 1 A. IF staining of Nestin at day 15 as NCC marker, scale bar = 100 µm. B. PAX3 vs. PAX6 gene expression to determine NCC vs. ectoderm. (N = 3) A paired t-test was performed to calculate significant differences; *p<0.05.*


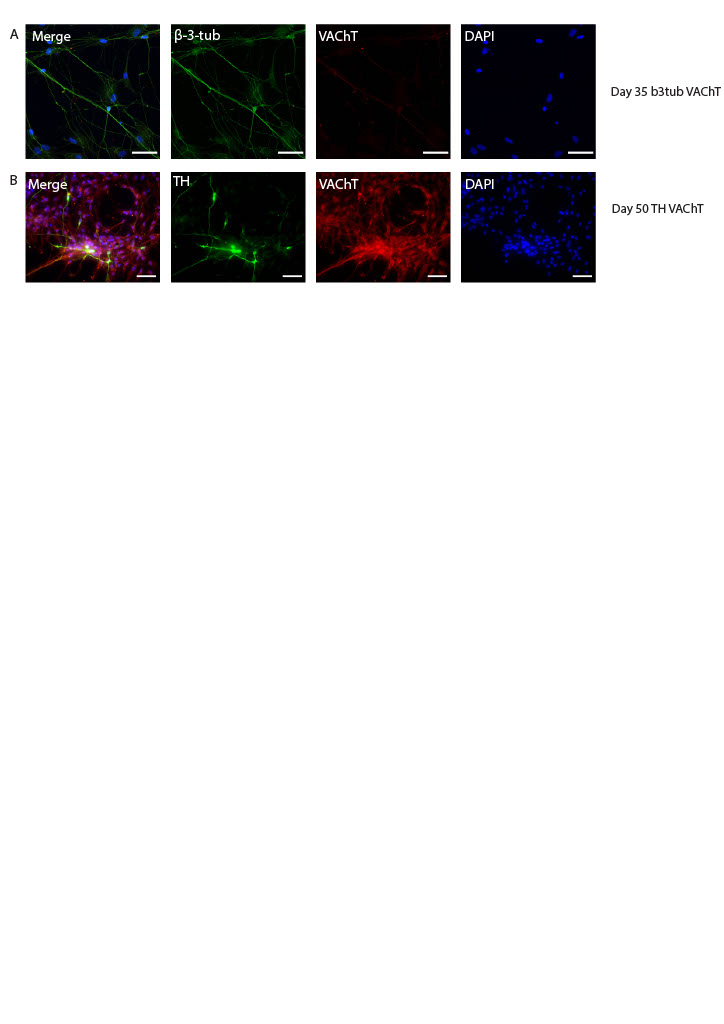


*Supplementary Figure 2 IF staining of hPSC-derived peripheral cholinergic neurons A. β-3-tubulin-VAChT double staining at day 35. At day 35 VAChT is not present yet in β-3-tubulin^+^ neurons. B. VAChT-TH double staining at day 50. Despite high TH expression in RNA, VAChT is predominant in the generated neurons.*


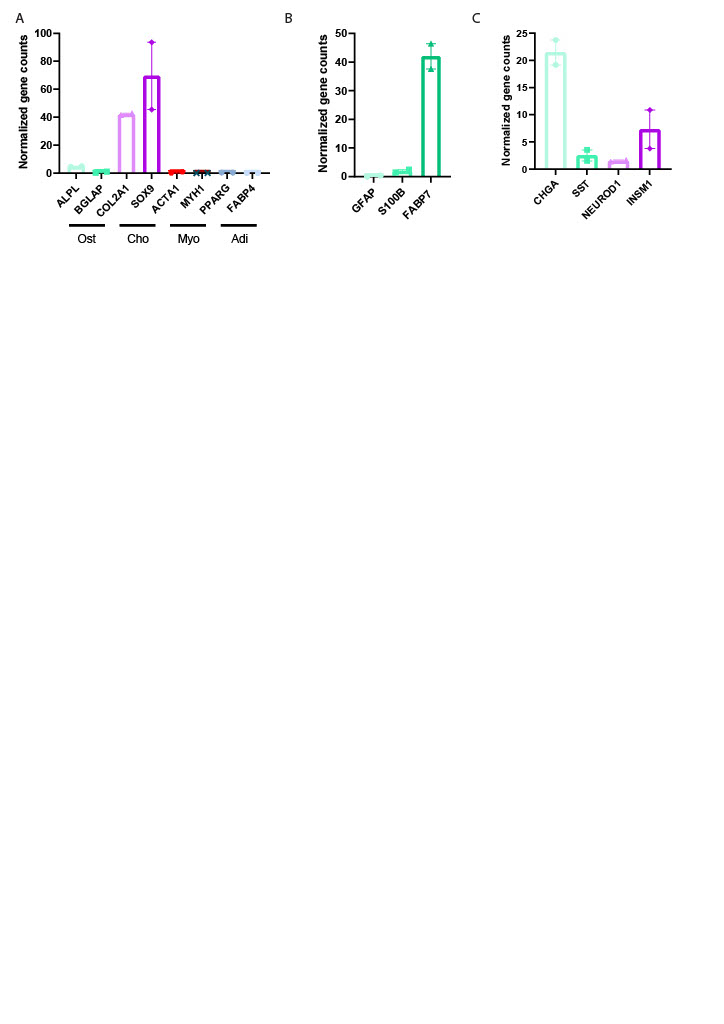


*Supplementary Figure 3 RNAseq showing expression of non-neuronal markers that originate from NCCs. A. Expression of mesenchymal cell markers. Ost = osteocytes; Cho = chondrocytes; Myo = myocytes; Adi = adipocytes. B. Expression of glial cell markers. C. Expression of endocrine cell markers.*

**Supplementary Figure 4**. *MEA analysis of neurons. Neurites extend over the electrodes of the MEA plate, and started spontaneous firing after day 50 of differentiation and increased up to day 73.*

***
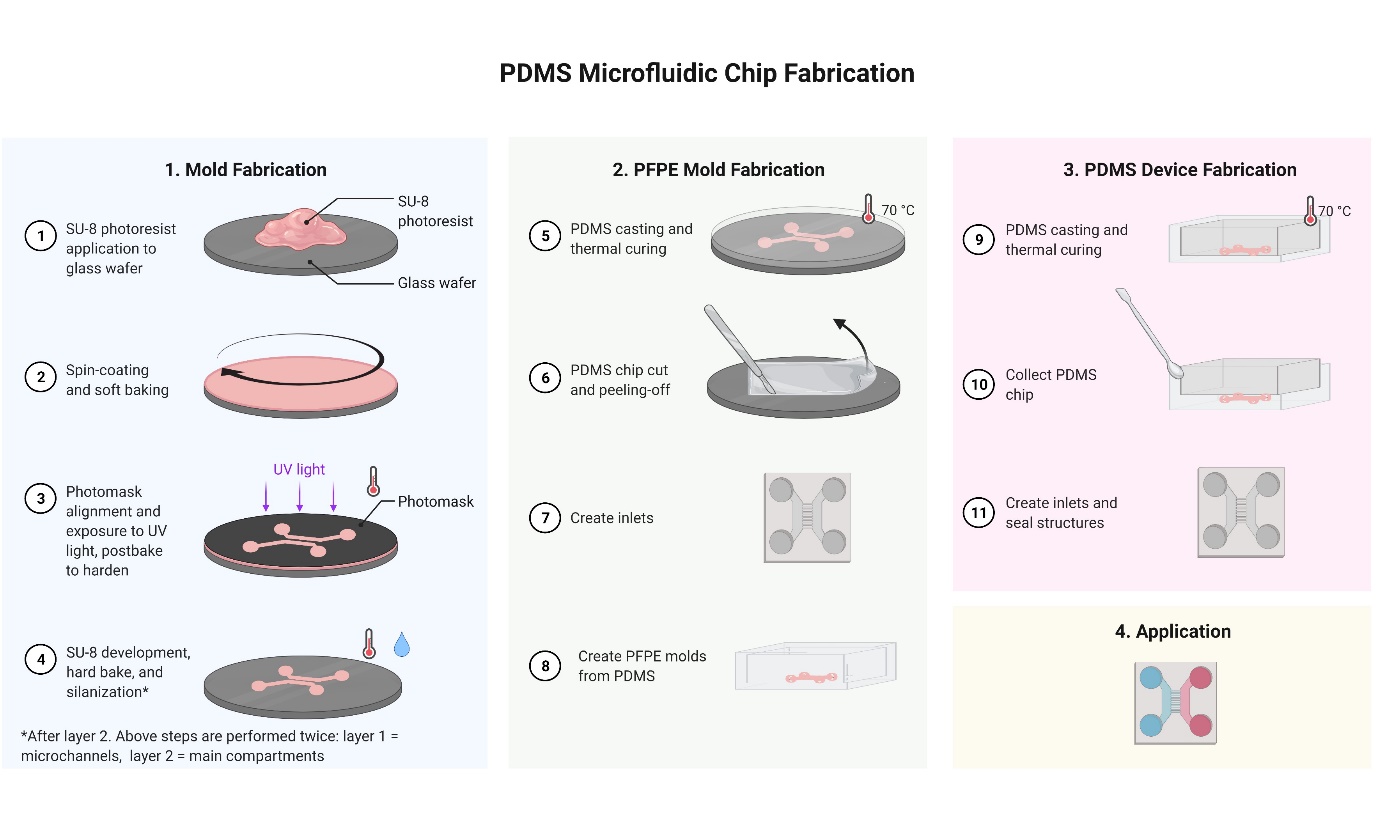
***

***Supplementary Figure 5.*** *Schematic overview of chip fabrication.* ***Left panel****: SU-8 structures on a glass wafer. A 3 µm thick layer of SU-8 was spin-coated onto a glass wafer, followed by a soft baking step. The wafer was covered by a chrome mask with microchannel structures and exposed to UV light. A postbake step was applied to harden the crosslinked SU-8. Non-exposed SU-8 was rinsed off using SU-8 developer. A next layer of two main compartments was added; the above steps were repeated with a 50 µm thick layer of SU-8. After the hard bake, a silanization step was applied.* ***Middle panel****: The glass wafer with SU-8 structures was placed in a petri dish, and a layer of 2-3 mm PDMS was added and placed on a hotplate for 70 min at 70°C. PDMS was peeled of and cut into squares around the structures. Inlets were created using a 8-mm-puncher. A PFPE mold was created around the PDMS square.* ***Right*** ***panel****: PDMS was added to the PFPE molds. PDMS was cured for for 70 min at 90°C. PDMS chips were collected from the mold and cut if necessary. Inlets were punched if required. Channels were sealed with a glass coverslip using oxygen plasma and filled with UP water immediately, before application. The final height was 65 µm ± 1 µm for the main compartments and 3 µm ± 0 µm for the microchannels, and the total volume of both main compartments and microchannels harbored 1.97 µL ± 0.03 µL. The medium reservoirs had a capacity of 89-143 µL each, depending on the thickness of the chip, and where filled up to a maximum of 100 µL. Created with BioRender.com.*


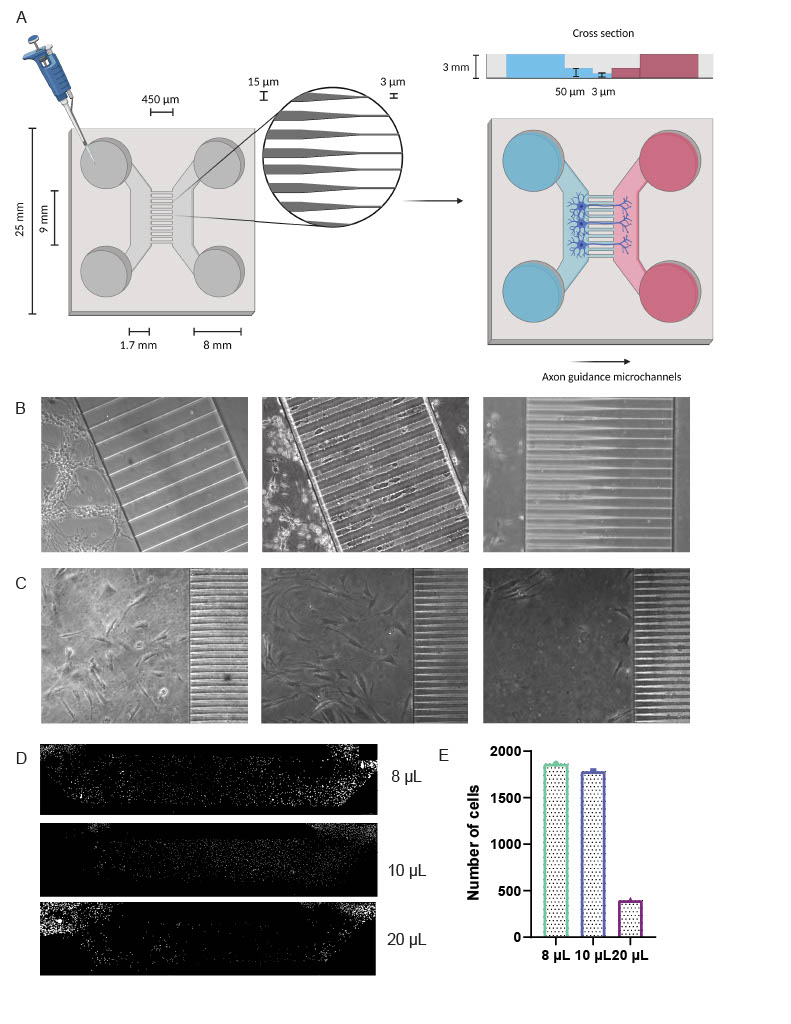


***Supplementary Figure 6****. Chip optimization. A schematic overview of the chip structure and dimensions. The chip was 25x25 mm, with two main compartments, each consisting of a 9 mm long channel, 1.7 mm wide and 50 µm high. The two compartments were connected by microchannels: 450 µm in length, 3 µm high. Medium reservoirs (8 mm Ø) were inserted at the four corners. During application, both main compartments can harbor cells in their preferred medium, as the medium does not mix if hydrostatic pressure is in equilibrium. Created with BioRender.com. B. Optimization of microchannel dimensions. Left: 3 µm x 3 µm (Xona Microfluidics®) channels did not allow cells to travel between compartments and were too narrow for axons to enter the microchannels. Middle 10 µm x 10 µm: SH-SY5Y cells migrated into the microchannels, proliferating in both main compartments. Right: Tapered channels 15 µm to 3 µm, 3 µm high. The microchannel inlet allowed both axons and cells to go in, but 3 µm outlet only allowed axons to reach the axonal compartment. C-E Optimization of cell loading volume. A higher volume corresponds to a higher velocity of cells when entering the main compartment, and thus less attachment here. C. Left: 8 µL of suspension 50.000 SH-SY5Y cells; center: 10 µL of suspension 50.000 SH-SY5Y cells; right: 20 µL of suspension 50.000 SH-SY5Y cells. D. DAPI staining of SH-SY5Y cells in the main compartment. Upper: 8 µL, center: 10 µL, lower: 20 µL. E. Quantification of DAPI staining 8 and 10 µL of cell suspension added to the chip led to 4x more cells in the channel compared to 20 µL.*


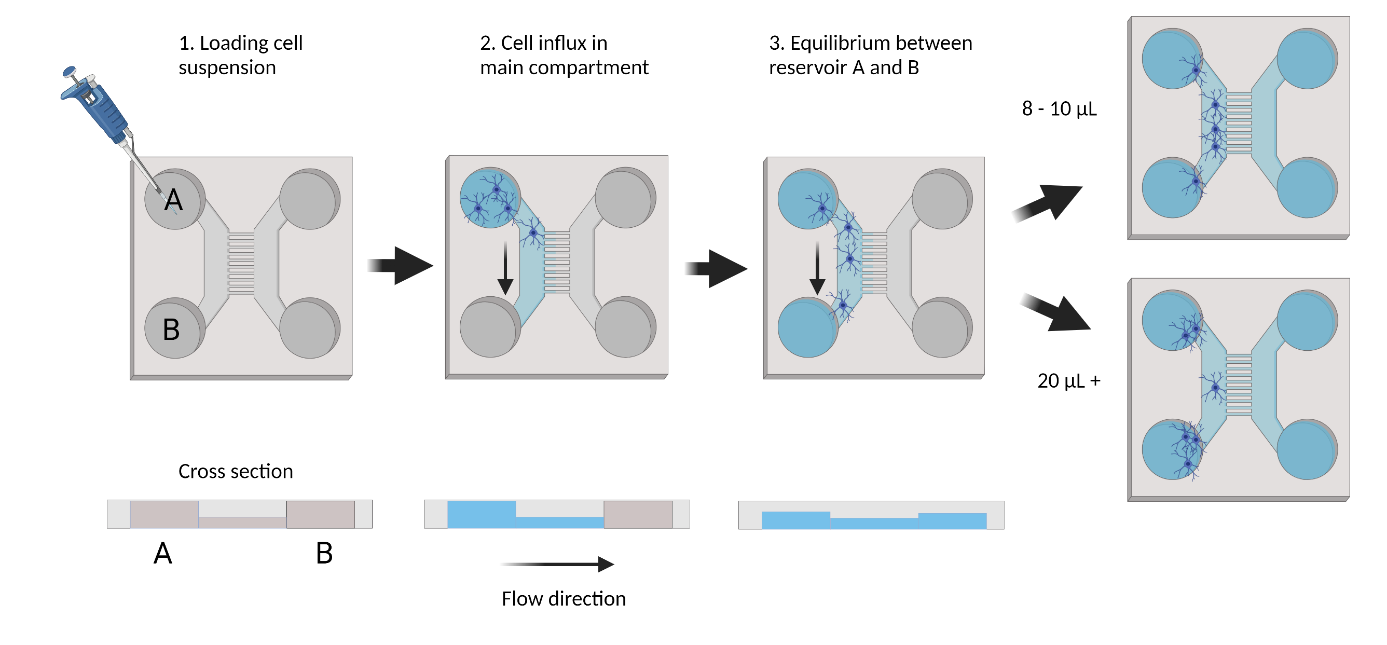


***Supplementary Figure 7****. Cell loading of different volumes*. Cells are seeded via the medium reservoirs while all reservoirs are empty. Add x μL of cell suspension to reservoir A (1), this creates an imbalance in equilibrium and allows cells to flow in, from reservoir A in the direction of reservoir B (2); the added cell volume is the only volume that disturbs the equilibrium. Thus, a higher volume resulted in a higher velocity of fluid and cells when entering the main compartment to establish equilibrium again. A lower cell velocity is necessary to attach to the main compartments. Finally, the equilibrium between reservoir A and B is re-estated and cells continue to settle (3). When the equilibrium was more off-balance, the rapid influx resulted in cells flowing into compartment B without attaching in the main compartment. Note that because of balance, half of the cell suspension will always remain in compartment A. Created with BioRender.com.

Supplementary Table 1 Stages of airway neuronal differentiation.

| Timepoint | Stage | Frequency change/week | medium | Comments |
| --- | --- | --- | --- | --- |
| Day -0 | Pluripotency | 7 | mTESR1 |  |
| Day 0-12 | Vagal NCC induction | 3-4 | KSR/N2 supplemented with SB431542 , LDN193189, CHIR99021, and r.a.  Day 0: 100% KSR + SB + LDN  Day 2: 100% KSR + SB + LDN + CHIR  Day 4: 75% KSR + 25% N2 + SB + LDN + CHIR + r.a.  Day 6: 50% KSR + 50% N2 + SB + LDN + CHIR + r.a.  Day 8: 25% KSR + 75% N2 + SB + LDN + CHIR + r.a.  Day 10: 100% N2 + SB + LDN + CHIR + r.a. | Day 0 – Day 4: 2 mL medium/well of 6 well plate.  Day 6 – Day 8: 2 mL medium/well of 6 well plate.  Day 10: 5.5 mL medium/well of 6 well plate. |
| Day 12-16 | Spheroid stage | 3-4 | VP speriod medium + FGF2 + CHIR |  |
| Day 16-30 | Neuronal induction | 3 | AN medium + BDNF + a.a. |  |
| Day 30-40 | Neuronal induction/maturation | 2 | AN medium + BDNF + a.a. (+ FB + LM) | From day 30: 1.5x medium volume  From day 35: Add ASM to co-cultures; add FB/LM to medium |
| Day 40-50 | Neuronal maturation | 1 | AN medium + BDNF + a.a. + FB + LM | From day 45: add dexamethasone to medium |

Supplementary Table 2 Medium compositions

| Medium | Composition |
| --- | --- |
| mTeSR1 medium for maintenance | - mTeSR1 basal medium (STEMCELL technologies, Vancouver, Canada, Cologne, Germany, 85850) - 20% mTeSR1 supplement (STEMCELL technologies, Vancouver, Canada, Cologne, Germany, 85850) - 1% penicillin/streptomycin (p/s 5000 units, Thermo Fischer, Waltham, US, 1989509) |
| KSR medium | - Knockout DMEM (Life Technologies, 10829-018) - 15% KSR (Life Technologies, 10828-028) - 10 µL mL^-1^ L-glutamine (200 mM, Life Technologies, 25030-081) - 10 µL mL^-1^ MEM Non-Essential Amino Acids (Thermo Fischer, Waltham, US, Waltham, US, 11140-050) - 55 µM of 2-mercaptoethanol (Life Technologies, 21985-023) - 1% p/s (5000 units) |
| N2 medium | - DMEM:F12 HEPES (Thermo Fischer, Waltham, US, Waltham, US, 11574546) - 20 µL mL^-1^ N2 supplement (Thermo Fischer, Waltham, U.S., A1370701) - 1% p/s (5000 units) |
| VP spheroids medium | - Neurobasal® medium (Thermo Fischer, Waltham, U.S., 21103049) - 10 µL mL^-1^ N2 supplement - 20 µL mL^-1^ B27 supplement (Thermo Fischer, Waltham, US, 17504044) - 10 µL mL^-1^ Glutamax (Thermo Fischer, Waltham, US, 35050061) - 10 µL mL^-1^ MEM Nonessential Amino Acids - 10 ng mL^-1^ FGF2 (Thermo Fischer, Waltham, U.S., PHG6015) - 3 µM CHIR 99021 |
| AN medium | - Neurobasal® medium - 10 µL mL^-1^ N2 supplement   20 µL mL^-1^ B27 supplement   - 10 µL mL^-1^ Glutamax - 10 µL mL^-1^ MEM Nonessential Amino Acids - 100 µM ascorbic acid (Sigma-Aldrich, A5960) - 10 nL mL^-1^ BDNF (Peprotech, 450-02) - 2 µg mL^-1^ LM (from day 35) - 2 µg mL^-1^ FB (from day 35) |
| ASM medium | - DMEM (ThermoFisher, 11965084) - 10% FBS, - 2% p/s (10.000 units) - 0,6% Amphotericin B (ThermoFisher, 15290018) |

Supplementary Table 3 Normalized gene counts is provided as a separate Excel Table.

Supplementary Table 4 List of the primers used for PCR analysis for human genes

| Gene | Forward primer | Reverse primer |
| --- | --- | --- |
| SDHA | TGCAGAAGGTGCGGATTGAT | TCCAGAGTGACCTTCCCAGT |
| RPL13A | TCGTACGCTGTGAAGGCATC | GCTTTTTCTTGTCGTAGGGGG |
| POU5F1 (OCT4) | GGCCACACGTAGGTTCTTGA | GCTGAATACCTTCCCAAATAGAACC |
| Nanog | CAATGGTGTGACGCAGAAGG | TGCACCAGGTCTGAGTGTTC |
| PAX3 | CGCTTCCTCCAAGCACTGTA | AGAGCGCGTAATCAGTCTGG |
| PAX6 | ACGTGGGAGAAGTTGGAATCT | CGCGCCCCTAGTTAAAGTCT |
| HOXB3 | ACTACCAGCCCCCTCTCAAA | CCCCGTTGTAGTCCAGGTTC |
| HOXB5 | CCGGCTCTTACGGCTACAAT | GGCCTCGTCTATTTCGGTG |
| PHOX2B | GTACGCCGCAGTTCCTTACA | CTCCTGCTTGCGAAACTTGG |
| EDNRB | GGTGGTCTCTGTGGTTCTGG | CCACTTCCCGTCTCTGCTTT |
| ASCL1 | CAAGCAAGTCAAGCGACAGC | CTCATCTTCTTGTTGGCCGC |
| TUBB3 | GGAGATCGTGCACATCCAGG | GCCCCACTCTGACCAAAGAT |
| BDNF | GCCTTTGGAGCCTCCTCTTC | CCCTGCAGCCTTCTTTTGTG |
| NGF | GCAAGCGGTCATCATCCCAT | AAAGGTGTGAGTCGTGGTACA |
| NTF3 | AATTACCAGAGCACCCTGCC | GTACTCCCCTCGGTGACTCT |
| NTF4 | CTTTCGGGAGTCAGCAGGTG | GCCACCTTCCTCAGCGTTAT |
| GDNF | GAGGAAAAGGTCGGAGAGGC | GAGCCGCTGCAGTACCTAAA |
| NGFR | CCGACAACCTCATCCCTGTC | CCACTGTCGCTGTGGAGTTT |
| NTRK1 | TCTGCACTGTTCTTGTGCCC | AGACCCCCAGATTTCATCACC |
| NTRK2 | TGACGATGGTGCAAACCCAA | CTGGGCCTTTCATGCCAAAC |
| NTRK3 | GAGCCCTTTCCAGTTGACGA | TCACCACTGATGACAGCCAC |
| ChAT | TTTTGTGAGAGCCGTGACTG | CACAGGACCATAGCAGCAGA |
| NOS1 | CTGGTCCTCAGCAAGGGTTT | ATGTCTGGGGAGGAGCTGAT |
| VIP | CCAGGCATGCTGATGGAGTT | CCCTCACTGCTCCTCTTTCC |
| TH | GGGCTGTGTAAGCAGAACG | AAGGCCCGAATCTCAGGCT |
| PRPH | GCCTGGAACTAGAGCGCAAG | GTACTTGGACTTGTACCACT |
| ACTA2 | GGCAAGTGATCACCATCGGA | GTGGTTTCATGGATGCCAGC |
| CHRM3 | GATACACAGCCCCTCCGATG | GGTATGACCTCCCAGAGGGT |

Supplementary Table 5 Antibodies

| Antibody | host | Dilution | supplier |
| --- | --- | --- | --- |
| Anti-β-3-tubulin | Mouse monoclonal | 1:200 | Santa Cruz Biotechnology, sc-51670 |
| Anti-OCT4 | Mouse monoclonal | 1:200 | Santa Cruz Biotechnology, sc-5279 |
| Anti-SOX10 | Mouse monoclonal | 1:50 | Santa Cruz Biotechnology, sc-365692 |
| Anti-SYP | Mouse monoclonal | 1:50 | Santa Cruz Biotechnology |
| Anti-VAChT | Guinea Pig | 1:200 | Synaptic Systems, 139105 |
| Anti-peripherin | Mouse monoclonal | 1:200 | Santa Cruz Biotechnology, sc-377093 |
| Anti-PGP | Rabbit | 1:200 | GeneTEx, GTX109637 |
| Anti-TH | Rabbit | 1:1000 | Sigma, AB152 |
| PE-CD271 (p75-NGFR) | Anti-Human | 1:50 | Biolegend, 345101 |
| Alexa Fluor® 647 CD57 (HNK1) | Anti-Human | 1:50 | Biolegend, 359613 |
| PE Anti- β-3-tubulin | Anti-Human | 1:1000 | BioTechne, NB600-1018PE |
| APC Anti-Choline Acetyltransferase | Anti-Human | 1:5000 | Abcam, ab224001 |
| Cy™3 AffiniPure | Donkey-anti-Mouse | 1:200 | Jackson ImmunoResearch, 715-165-150 |
| Alexa Fluor 488 | Donkey-anti-Mouse | 1:1000 | Invitrogen, A-21202 |
| Alexa Fluor 488 | Donkey-anti-Rabbit | 1:1000 | Invitrogen, A-21206 |
| Cy™3 AffiniPure | Donkey-anti-Guinea Pig | 1:200 | Jackson ImmunoResearch, 706-165-148 |
